# Surface complexation and multilayer formation in the adsorption of NADA and phosphate on magnetic iron oxide nanoparticles: implications for bioseparation

**DOI:** 10.1039/d6na00243a

**Published:** 2026-06-25

**Authors:** Paula Fraga-García, Carlos Eduardo Díaz-Cano, Spartak S. Khutsishvili, Vanessa Jurado-Davila, Lucía Abarca-Cabrera, Jozef Lengyel

**Affiliations:** a Technical University of Munich (TUM), School of Engineering and Design, Department of Energy and Process Engineering, Chair of Bioseparation Engineering Boltzmannstraße 15 Garching 85748 Germany p.fraga@tum.de; b Georgian American University, School of Medicine 10 M. Aleksidze St. 0160 Tbilisi Georgia; c Ivane Javakhishvili Tbilisi State University, Rafael Agladze Institute of Inorganic Chemistry and Electrochemistry 11 E. Mindeli St. 0186 Tbilisi Georgia; d Technical University of Munich (TUM), School of Natural Sciences, Department of Chemistry, Chair of Physical Chemistry Lichtenbergstraße 4 Garching 85748 Germany

## Abstract

Recent decades have seen major advances in industrial production using biological systems. However, downstream processing has not kept pace with increasing productivity and remains a bottleneck in biotechnological production. Magnetic separation offers promising opportunities for bioseparation, but broader application requires a deeper understanding of the capture step. Here, we investigate the adsorption of two small, highly soluble molecules onto bare iron oxide nanoparticles (BIONs). The amino acid derivative *N*-acetyl-l-2,4-diaminobutyric acid (NADA) forms more than one layer on the BION surface with a saturation capacity of 262 mg g^−1^ that decreases to about 164 mg g^−1^ for less pure samples. The anion phosphate, measured at lower concentrations in solution, remains below monolayer coverage (12 and 14 mg g^−1^ in water and artificial sea water, respectively) and at high ionic strength shows a significant decrease in capacity after apparent saturation. Ferromagnetic resonance measurements of the phosphate-BIONs in water indicate shielding of BION–BION interactions due to the phosphate layer. The shape of the adsorption isotherms, together with infrared (IR) spectroscopy, suggest that multiple adsorption mechanisms govern particle loading as solute concentration increases. IR data point to the formation of inner-sphere phosphate-surface complexes, which persist even when a NADA layer is pre-adsorbed onto the BION surface. In contrast, NADA does not adsorb onto the BIONs from a microalgae lysate, where other biomolecules preferentially occupy the nanoparticle surface. Overall, these results contribute to a better understanding of adsorption processes on solid carriers and provide valuable guidance for designing nanoparticle-based systems capable of capturing and releasing (bio)molecules in bioseparation and related applications.

## Abstract 2

NADA strongly accumulates on nanoparticles unless lysate is present, while phosphate forms persistent inner-sphere complexes that shift in saline conditions.

## Graphical abstract

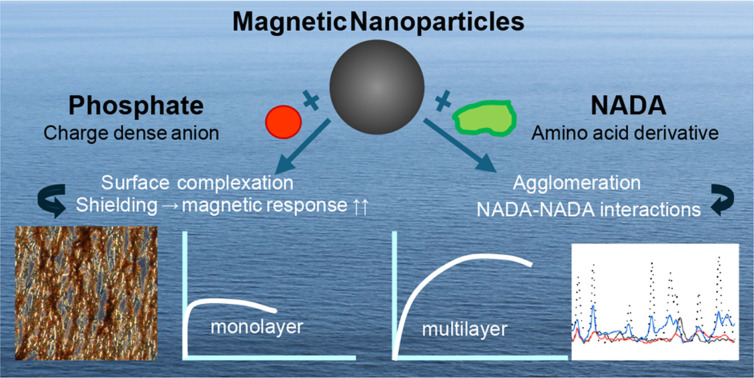

## Introduction

According to Farjadian *et al.*^1^ nanotechnology emerged as a widely recognized concept in the 1980s, although its intellectual roots date back to the late 1950s, when Richard Feynman envisioned manipulating matter at the atomic scale. Since then, the field has advanced markedly and now plays an important technological role. In this context, bionanotechnology has emerged as a particularly promising branch with applications in biomedicine^2,3^ and largely unexplored potential in industrial bioprocessing.^2,4^

Nanomaterials offer a large surface-to-volume ratio that makes them suitable for molecule capture. In particular, magnetic nanomaterials are promising carriers for bioseparation and biofractionation because their magnetic susceptibility enables rapid and efficient separation processes.^5^ While biotechnological upstream processing has advanced considerably in recent decades, the lack of low-cost and efficient purification methods remains a major bottleneck for industrial biotechnology.^6–9^ Magnetic separation can help address this challenge. Its core step is molecular capture, which is primarily driven by adsorption. However, adsorption and partitioning mechanisms in solid–liquid systems remain difficult to predict, which limits the ability to design optimal separation processes. Better understanding of these mechanisms would enable the rational design of particle surfaces and expand the flexibility of adsorption-based separation techniques.

While many studies in biomedicine and bionanotechnology investigate the adsorption of large biomolecules such as proteins onto nanoparticles,^10,11^ relatively few focus on small molecule adsorption.^12^ Such studies are more common in soil chemistry, where solid phases are typically larger and less homogeneous. In this work, we compare the adsorption behaviour of two small molecules with very different charge characteristics when incubated with bare iron oxide nanoparticles (BIONs): the biotechnologically produced amino acid derivative *N*-acetyl-l-2,4 diaminobutyric acid (NADA) and the inorganic anion phosphate. Our goal is to gain further insight into the adsorption behaviour of biologically relevant small molecules, while exploiting the advantages of BIONs for large-scale applications, namely, low cost and simple synthesis.

NADA is an acetylated derivative of the non-canonical amino acid l-2,4-diaminobutyric acid. According to Martin, NADA belongs to the class of compatible solutes, defined as “water-soluble, low molecular weight, and neutrally net-charged molecules, which are accumulated by halophilic organisms to preserve the functionality of the cellular components in the saline environment”.^13^ This acetylated amino acid, used by bacteria to compensate for osmotic pressure,^13^ has a molecular weight of 160 g mol^−1^,^13^ is zwitterionic,^13,14^ and carries no net charge at neutral pH.^14^ It also has potential applications in the cosmetic and healthcare industries.^13,14^

Phosphate, in contrast, is a common anion in aqueous environments (*M*_w_ ≈ 95 g mol^−1^) that is partially or fully dissociated over a broad pH range. It is a charge-dense, kosmotropic ion capable of inducing protein precipitation.^15^ Phosphate groups are ubiquitous in biomolecules, serving as structural and functional units in nucleic acids, phospholipids, nucleotides, and phosphorylated proteins.^16^ They also exhibit strong affinity for metal oxides and functionalized surfaces, and phosphate buffers are widely used in biotechnological media. In general, the phosphorus cycle is gaining attention due to limited resources, and its importance for crop production.^16,17^ Additionally, phosphate is the most common form of phosphorous in nature.^18^

Both NADA and phosphate are highly soluble in water (≥100 mg L^−1^)^13^ and therefore strongly water affine. Despite similar solubility, their adsorption behaviour is expected to differ due to their distinct charge properties and solution chemistry. We therefore investigated their adsorption experimentally. Moreover, we analysed their behaviour in sequential adsorption experiments and in the presence of a complex biological system, namely a microalgal lysate. These experiments follow our broader strategy of studying simultaneous and sequential processes to better understand the competition and cooperation effects in adsorption systems.^19,20^

To approach realistic applications in the separation of complex biological mixtures, we also examined whether the pre-adsorption of NADA or phosphate affects the subsequent adsorption of the other species. Similar studies with proteins and mixtures have shown that an existing protein corona does not prevent further adsorption and exchange processes.^20–22^ Here, we investigate whether highly soluble molecules such as NADA and phosphate behave similarly. Phosphate is of particular interest due to its strong negative charge and its suitability as an eluting agent, as shown previously.^23,24^ NADA, by contrast, is a small organic molecule that is zwitterionic but carries no net charge at neutral pH. Thus, this study compares two small molecules with similar solvent affinity, but different charge characteristics and explores their potential role as co-solutes that influence adsorption and release of larger biomolecules in bioseparation processes.^19,24^ One long-term goal of our work is the large-scale application of magnetic nanoparticles for bioseparation processes. These nanoparticles are very beneficial due to their magnetic susceptibility and high surface-to-volume ratio. Achieving this goal requires a more systematic understanding of adsorption mechanisms under different conditions.

## Experimental section

### Biological materials

The compatible solute *N*-acetyl-l-2,4-diaminobutyric acid (NADA), obtained from saline fermentation of *Halomonas elongata*, is a precursor molecule in the ectoine biosynthesis pathway and serves as a protective agent.^14^ Details on the NADA production, separation, and purification processes are described elsewhere.^13^ For experiments with impure NADA, a powder with 73% purity (Bitop AG, Witten, Germany) was used. The sample (lot 17NADA0601) is composed of approximately 72% γ-NADA, 1% α-NADA, and 27% NaCl.^13^ Pure NADA was obtained from this material by crystallization in ethanol.^13,14^

The *Microchloropsis salina* cultures (SAG 40.85), previously known as *Nannochloropsis salina*, were provided by the Chair of Biochemical Engineering at the Technical University of Munich. The strain was originally obtained from the Culture Collection of Algae at the University of Göttingen. The algae were cultivated in modified artificial seawater (ASW)^25^ in open thin-layer cascade photobioreactors.^26^ The batch used in this study had a biomass concentration of 7.7 g L^−1^ (dry weight). The initial protein concentration, determined using a bicinchoninic acid (BCA) assay, was approximately 3 g L^−1^ (≈39% of the biomass). The algae suspension had a pH of 7.85 and a conductivity of 38.5 mS cm^−1^ and was stored at 4 °C. The ASW medium consisted of a complex mixture of salts (see Table S1 for composition).

### Magnetic nanoparticles

The BIONs were synthesized by co-precipitation of Fe^2+^ and Fe^3+^ aqueous salt solutions in an alkaline environment. Briefly, 200 mL of ferrous chloride (100 mmol FeCl_2_·4H_2_O, Bernd Kraft GmbH) and ferric chloride (200 mmol FeCl_3_·6H_2_O, AppliChem) were mixed with 500 mL sodium hydroxide solution (1 mol NaOH, AppliChem) in degassed double-deionized water (dd-H_2_O) in a stirred tank reactor under a nitrogen atmosphere. After synthesis, the suspension was washed several times with degassed dd-H_2_O. The BIONs were stored as a suspension in dd-H_2_O at a concentration of 10 g L^−1^ at room temperature (pH 7.8, conductivity 179 µS cm^−1^).

Detailed synthesis and characterization of the nanoparticles are described elsewhere and summarized here for completeness.^20^ The nanoparticles had a specific surface area of 78 m^2^ g^−1^ as obtained from nitrogen adsorption (BET measurements). The mean particle size was 9.2 nm, based on XRD measurements, and 8.9 nm, based on TEM analysis. Magnetization measurements yielded a value of ∼70 Am^2^ kg^−1^ and FTIR data confirmed the presence of the common bands for superparamagnetic iron oxide nanoparticles.

Ferromagnetic resonance (FMR) experiments were performed using a different batch of BIONs synthesized and characterized in the same way (see Abarca-Cabrera *et al.*^27^). These particles had a TEM size of 11.6 ± 3.2 nm, an XRD size of 7.6 ± 0.6 nm, and a specific surface area of 96.9 ± 0.2 m^2^ g^−1^.

### Analytical methods and experimental procedures

The dry weight of algae and BION samples was determined gravimetrically. For this purpose, 2 mL Eppendorf microcentrifuge tubes were dried overnight at 60 °C. Subsequently, 1 mL of *M. salina* and BION suspension was added to the tubes in quadruplicate. The samples were centrifuged at 10 000 g for 2 min using a Heraeus Fresco 17 centrifuge (Germany), and the supernatants were discarded. To remove residual salts, 1 mL dd-H_2_O was added, followed by vigorous mixing using alternating vortexing and centrifugation steps. After removal of the supernatant, the samples were dried again overnight at 60 °C. The dry weight was determined once a constant weight was achieved.

To disrupt the *M. salina* cells, 1 mL of glass beads (0.1–0.25 mm, Retsch GmbH) was mixed with 1 mL of algae suspension in 2 mL Eppendorf tubes. The sample were milled for 15 min at a frequency of 25 s^−1^ using a Mixer Mill MM400 (Retsch GmbH, Germany). Approximately 700 µL of lysate was recovered from the supernatant of each tube, and the remainder was discarded. Cell disruption was performed immediately prior to each set of experiments using the algae lysate.

Protein concentrations were quantified using the bicinchoninic acid (BCA) assay which utilizes the reduction of Cu^2+^ to Cu^+^ by proteins to a purple-coloured complex in alkaline environment, detectable spectrophotometrically at 562 nm. Bovine serum albumin (BSA) was employed as standard protein. Calibration curves were prepared from 2 g L^−1^ BSA stock solutions in ASW medium. The protein concentrations were measured in triplicate in Nunc 96-well microplates (ref. 260836, Thermo Fisher Scientific) in a TECAN Infinite M200 plate reader (Tecan Deutschland GmbH, Germany). For each measurement, 25 µL of sample were mixed with 200 µL of working reagent and incubated at 30 °C for 30 min. Concentrations were calculated using a 9-point calibration curve after blank subtraction. To quantify the protein adsorbed onto BIONs, the particle suspensions were diluted to approximately 1 g L^−1^. A 25 µL aliquot of particle suspension was mixed with 200 µL of working reagent and incubated at 30 °C for 30 min. The samples were then transferred to 0.22 µm filter well plates and centrifuged at 4000 rpm for 10 min using a Heraeus Megafuge 16R centrifuge (Thermo Scientific, Germany). Total protein concentrations were determined by BCA assay from both the supernatants and the BIONs fractions after magnetic separation.

Orthophosphate concentrations were determined according to the DIN EN ISO 6878.2004 method^28^ previously used in our group.^29^ Phosphate is detected *via* formation of a phosphomolybdenum blue complex in the presence of antimony and ascorbic acid, similar to the method reported by Murphy and Riley.^30^ For quantification, 500 µl of sample was mixed with 10 µl of 10% (w/v) ascorbic acid solution, 20 µl molybdate reagent, and 470 µl dd-H_2_O (total volume of 1 mL). The molybdate reagent was prepared by dissolving 3.25 g ammonium heptamolybdate tetrahydrate in 25 mL dd-H_2_O and 0.0875 g of potassium antimonyl tartrate in a separate 25 mL dd-H_2_O. Subsequently, 75 mL of 50% sulfuric acid was slowly added to the ammonium molybdate before mixing with the potassium antimonyl tartrate solution. After reagent addition, samples were incubated at 30 °C for 30 min and absorbance was measured at 835 nm.

Although the DIN method recommends detection at 880 nm,^28^ measurements at 835 nm showed improved linearity in our system. Phosphate concentrations were determined using a 7-point calibration curve (0.5–16 mg L^−1^). Calibration standards were prepared before each measurement from a 1 g L^−1^ PO_4_ Certipur® standard solution (Merck, Germany) diluted in dd-H_2_O or ASW. The quantification protocol was optimized for measurements in water and in ASW. Experimental conditions were selected to maintain near neutral pH, while minimizing the addition of the other ions to avoid changes in ionic strength. All experiments were conducted at pH 6–8, where H_2_PO_4_^−^ and HPO_4_^2−^ are the dominant phosphate species. For simplicity, phosphate species are referred to as PO_4_ through this work.

NADA concentrations in the supernatants after adsorption experiments were determined using high-pressure liquid chromatography (HPLC) on a 1260 Infinity II system (Agilent, Germany) equipped with a YMC-Pack Polyamine II column.^13^ The column consisted of silica beads coated with polyamine groups (particle size 5 µm, pore size of 12 nm). A 20 µL sample was injected and separated at a flow rate of 1 mL min^−1^ using a mobile phase of 65% acetonitrile and 35% dd-H_2_O (both filtered through 0.22 µm cellulose membranes). Chromatograms were recorded at 198 nm over a runtime of 30 min. Peaks corresponding to α-NADA and γ-NADA (approximately 14 and 16 min of elution time) were integrated and summed to determine the total NADA concentration. Quantification was based on a 6-point calibration curve (0.05–0.5 g L^−1^) prepared from purified NADA standards. All samples were diluted to approximately 0.5 g L^−1^ prior to analysis. For each incubation condition, three independent experiments were prepared simultaneously (technical triplicates).

For experiments involving NADA in *M. salina* lysates, a second chromatographic method was used to confirm the results. In this case, a Luna HILIC column (Phenomenex) packed with cross-linked diol-modified silica particles (3.5 µm particle size, 20 nm pore size, surface area 200 m^2^ g^−1^) was employed. The mobile phase consisted of 80% acetonitrile, 10% dd-H_2_O, and 10% 100 mM ammonium formate (pH 3) at a flow rate of 1 mL min^−1^. Samples were analysed for 15 min with detection at 198 nm. Prior to analysis, all samples and standards were diluted in the same solvent mixture and filtered through 0.22 µm membranes.

UV-vis absorption spectra of the investigated compounds were recorded using a TECAN Infinite M200 plate reader. Measurements were performed in Greiner 96-well UV plates (Greiner Bio-One International GmbH, Germany) using 100 µL of sample solution.

Infrared spectra were recorded using a Brucker Alpha II Fourier-transform infrared (FTIR) spectrometer (Brucker, Germany). For each measurement, 2 µL of sample were deposited on the glass surface and dried with a hair dryer. Spectra were collected against atmospheric background with 24 scans in the range of 400–4000 cm^−1^ at a spectral resolution of 4 cm^−1^. Baseline correction was applied to all spectra.

Ferromagnetic resonance (FMR) measurements were performed on BIONs suspensions (10 g L^−1^) incubated with phosphate at three concentrations in dd-H_2_O (32 g L^−1^, 750 mg L^−1^, and 32 mg L^−1^). The suspensions were shaken for 1 h at room temperature prior to analysis. FMR spectra were recorded using a Bruker EMX FT X-band EPR spectrometer (9–10 GHz). Measurements were carried out using quartz capillaries (3 mm diameter) containing approximately 0.1 mg sample under the following conditions: modulation amplitude 10 G, modulation frequency 100 kHz, time constant 0.01 ms, conversion time 6 ms, microwave power 0.06325 mW, field range 5000 G/center field 3480 G, receiver gain 16 dB, and one averaged scan.

Microscopy images were obtained using an Axio Observer 7+ inverted light microscope (Zeiss, Germany). Isotherm samples were separated from the liquid phase, resuspended to 10 g L^−1^, and imaged at 20% illumination using a 305 Color Axiocam camera. Chlorophyll fluorescence at 630 nm was measured using a Colibri 7 LED light to verify adsorption of lysate components onto the BIONs. Images were processed using ZEN 2.5 Pro software.

Particle agglomeration sizes were determined by static light scattering (SLS) using a Laser Scattering Particle Analyzer (LA-950V2, Horiba, Japan). Samples were diluted in dd-H_2_O prior to measurement, and all measurements were performed in triplicate.

### Adsorption experiments

All adsorption experiments with NADA and phosphate were performed in 2 mL Eppendorf microcentrifuge tubes with a total volume of 1900 µL. Unless otherwise stated, 950 µL of BION suspension (10 g L^−1^) was mixed with 950 µL of the corresponding solute solutions, resulting in a final BION concentration of 5 g L^−1^. The mixtures were incubated for 1 h at 30 °C and 1000 rpm using a ThermoMixer® C (Eppendorf). After incubation, the particles were separated from the supernatants using a neodymium hand-magnet (0.5 T). The supernatants were collected and stored at −20 °C until analysis. The contacted particles were washed with 500 µL dd-H_2_O and resuspended to a concentration of 20 g L^−1^ (475 µL). Between measurements, samples were stored at 4 °C.

For the impure NADA isotherm, a 100 g L^−1^ NADA stock solution was prepared in dd-H_2_O (pH 7.5) and diluted (1 : 1) in sequential steps to obtain the required concentration range. The resulting solution had pH value between 7.4 and 7.8, and conductivities between 23.02 mS cm^−1^ and 161.90 µS cm^−1^. All NADA adsorption experiments were performed in technical triplicate.

Pure NADA solutions for the adsorption isotherm were prepared analogously. The initial NADA solution had a pH of 7.3 and was not adjusted to avoid increasing ionic strength. The final solutions had pH values between 6.88 and 7.74 and conductivities between 140 and 630 µS cm^−1^.

For the phosphate adsorption isotherm in water, solutions were prepared from a 1000 mg L^−1^ PO_4_ Certipur® standard solution (Merck, Germany) by stepwise dilution in dd-H_2_O. Ten solutions with concentrations between 2 and 750 mg L^−1^ were generated. These solutions had pH values between 7.07 and 7.42 and conductivities between 211 and 275 µS cm^−1^. Each PO_4_ adsorption condition was analysed in technical triplicate and measured in analytical triplicate.

Phosphate in saline media was studied only in the highly saline medium ASW, not in a lysate as with NADA. From previous experiments we knew that phosphate has an affinity for iron oxide and is a suitable molecule for eluting proteins from our BIONs.^23^ The focus was therefore on determining whether high ionic strength (as in ASW) would prevent interaction with the surface through charge shielding. For the PO_4_ adsorption isotherm in ASW, a concentrated phosphate stock solution (47.5 g L^−1^) was prepared by dissolving 4.67 g of K_2_HPO_4_ and 3.155 g KH_2_PO_4_ in 100 mL of dd-H_2_O (pH ≈ 7). This solution was diluted in modified ASW media lacking phosphate (pH 6.75) to obtain initial concentrations of 1500, 1000, and 512 mg L^−1^. Additional concentrations were generated by serial dilution in ASW, corresponding to the same concentration range as used in the water-based isotherm. The resulting solutions had pH values between 6.3 and 6.7 and conductivities between 41.8 and 40.4 mS cm^−1^.

Due to the limited availability of purified NADA, impure NADA was used to investigate NADA adsorption in *M. salina* lysates. The protein concentration of the lysate was determined using the BCA assay of the supernatant and confirmed by BCA analysis of the BION fraction. The measured protein concentration was approximately 3 g L^−1^, corresponding to about 39% of total algae biomass determined by dry weight. Two lysates were prepared: one supplemented with 3 g L^−1^ NADA (equivalent to the protein concentration in the lysate) and another containing 0.75 g L^−1^ NADA. Adsorption experiments were performed as described previously, using three BION-to-protein ratios of 0.25, 1 and 6 g_BION_ g_protein_^−1^. BION concentrations were adjusted by dilution or by concentration through magnetic separation and solvent removal.

Sequential adsorption experiments were also conducted to study the adsorption of NADA onto phosphate-coated BIONs and phosphate onto NADA-coated BIONs. For the initial coatings, BIONs were incubated under the same conditions used for the adsorption isotherms. NADA coatings were prepared using 12.5 g L^−1^ impure NADA, while phosphate coatings were prepared using 500 mg L^−1^ PO_4_ (Certipur standard solution). After incubation, supernatants were collected for concentration analysis, and the particles were washed twice with 500 µL dd-H_2_O (5 min incubation under isotherm conditions). Wash solutions were collected to quantify desorbed NADA or phosphate. The coated particles were stored at 4 °C prior to further experiments. Subsequently, NADA-coated BIONs were incubated with phosphate solutions (500 mg L^−1^ and 100 mg L^−1^), while phosphate-coated BIONs were incubated with NADA solutions (12.5 g L^−1^ and 3 g L^−1^) under the same adsorption conditions. Remaining solute concentrations were determined from the supernatants, and adsorption was qualitatively confirmed by FTIR analysis.

For lysate experiments, protein concentrations in the supernatants were measured using the BCA assay. Protein adsorption onto BIONs was calculated from the difference between the initial lysate concentration and the supernatant concentration after adsorption. The solids were then washed twice with distilled water, and the adsorbed proteins were also quantified directly from the particle phase using the BCA assay. For each condition, blank samples without nanoparticles were processed identically to account for background signals. All experiments were conducted in technical triplicate with analytical triplicates. Error bars represent the standard deviation of the resulting nine measurements.

## Results and discussion

As outlined in the introduction, the main goal of this work was to expand our understanding of (1) the adsorption of small molecules onto the BIONs and (2) the effect of sequential or simultaneous adsorption. In addition, we aimed to extend previous studies on corona formation from complex mixtures^20,31^ and from pure amino acids.^19^ By comparing an organic, zwitterionic molecule with an inorganic, negatively charged ion, we expect to gain further insight into adsorption mechanisms. The adsorbent here is the BIONs, whose properties are detailed in the Experimental Section: superparamagnetic iron oxide nanoparticles (*i.e.* high magnetization, but no remanence in the absence of a magnetic field) with a specific surface area of 78 m^2^ g^−1^ and a mean particle size of about 9 nm.

In this section we first present the adsorption behaviour of NADA and phosphate as individual components in water and in saline environments. The concentrations of the molecules remaining in the supernatant after adsorption were quantified, and adsorption onto the nanoparticle surface was qualitatively confirmed using FTIR measurements. Finally, more complex systems, *e.g.*, mixtures of NADA and phosphate and NADA in *M. salina* lysates, are investigated to provide further insight into adsorption processes in multicomponent systems involving BIONs.

### High saturation capacity for NADA

**Fig. 1:**
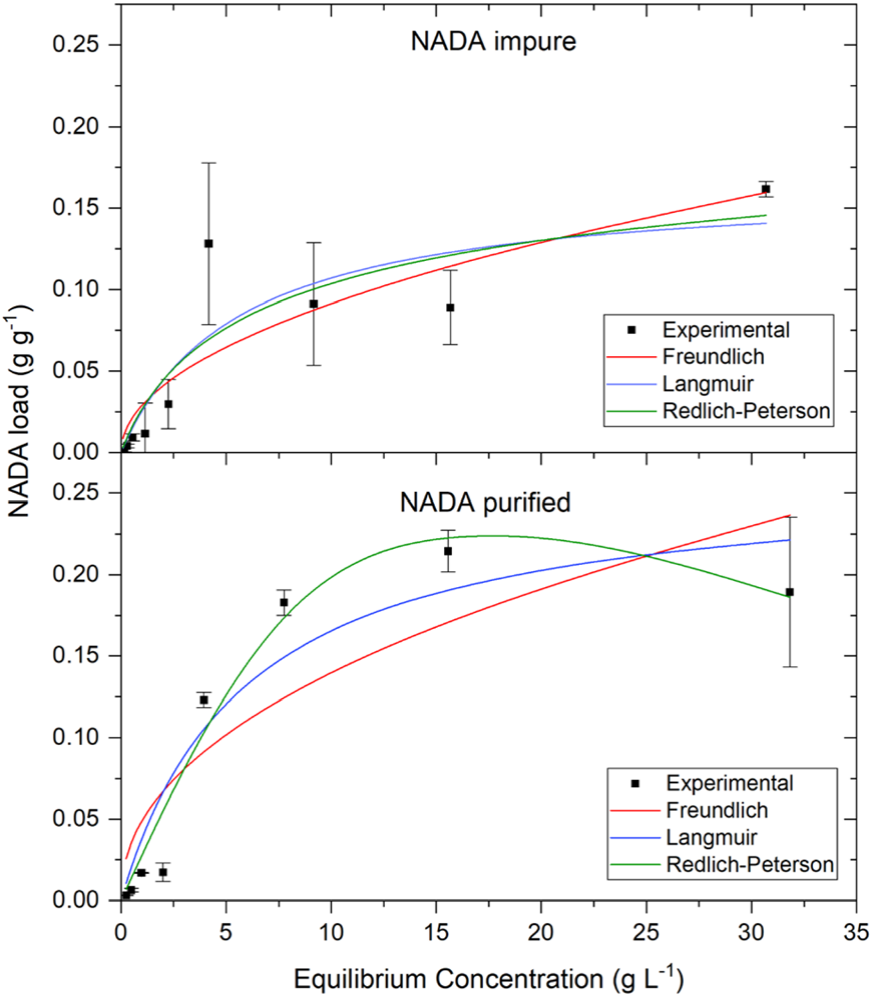
Adsorption isotherm of impure NADA (pH 7.4–7.83) and of purified NADA (pH 6.88–7.74) for a BION concentration of 5 g L^−1^. In the impure NADA isotherm, the data in the abscissa refer only to the NADA mass (without the mass of NaCl) in the sample powder. Data obtained using HPLC from UV absorbance at 198 nm from three individual incubation experiments. Additionally, the fits based on the Langmuir, Freundlich and Redlich–Peterson models are presented.

We first investigated the adsorption of the amino acid derivative NADA (see Fig. S1 for chemical structure), for which quantification *via* HPLC had previously been established in our group.^13^ We compared the adsorption isotherm of NADA from biotechnological production prior to the final purification, *i.e.*, in the presence of a high NaCl content, with the isotherm of highly purified NADA obtained by crystallization in our laboratory (purity >99%).^13,14^ Both adsorption isotherms are shown in Fig. 1. The concentration range studied was intentionally broad to approach the solubility limit of NADA in water, reported to be up to ∼0.6 M (slightly above 100 g L^−1^).^13^

Both isotherms exhibit remarkably high saturation capacity. The saturation value for impure NADA (164 mg g^−1^) is clearly higher than those observed for poorly soluble amino acids, such as tyrosine (>5 mg g^−1^),^19^ but slightly lower than those previously reported for very highly soluble amino acids, like proline (>300 mg g^−1^)^19^ and lysine (>250 mg g^−1^).^19^ The saturation capacity obtained for purified NADA is higher (262 mg g^−1^); pure NADA quantification shows for concentrations in solution <15 g L^−1^ smaller standard deviations than those of the impure samples. The differences may arise from the presence of NaCl in the impure system. Although NADA has an uncharged side chain, it remains zwitterionic due to its carboxyl and amine groups. The salt ions may therefore compete with NADA for adsorption sites on the BION surface.

The high saturation values of soluble amino acids, such as proline, valine (>200 mg g^−1^)^19^ and the amino acid derivative NADA further support the previously proposed relationship between adsorption capacity and solubility.^19^ Pušnik *et al.* reported adsorption capacities of 0.49 g and of 0.12 g lysine per gram BIONs, and suggested that amino acids adsorb in the form of multimolecular aggregates.^32^

Based on the measured capacities and the specific surface area of the BIONs (78 m^2^ g^−1^), we estimate that approximately seven NADA molecules nm^−2^ adsorb for impure samples and about eight molecules nm^−2^ for purified NADA. These values exceed typical monolayer coverages reported for amino acids on iron oxides.

For example, Costa *et al.*^33^ reported approximately 4.5 molecules nm^−2^ for a glycine monolayer on maghemite, while for phenylalanine intercalated between lipid molecules in a tightly packed lipid layer about 0.5 nm^2^ for one molecule were reported.^34^ Assuming that NADA occupies a similar surface area, *e.g.* about 3 molecules nm^−2^, the NADA saturation results suggest the formation of multilayers on the BION surface (approx. 60–70 mg g^−1^ for one layer), consistent with our previous observations for valine, proline, lysine and glycine.^19^

Comparable adsorption capacities have also been reported for peptides. Schwaminger *et al.*^35^ observed adsorption capacities of approximately 0.4 g g^−1^ for hexa- and octa-glutamic acid peptides on BIONs. Although such oligopeptides likely require only a single binding site per molecule, their larger molecular mass results in higher adsorbed mass compared with monomeric amino acids such as NADA. Adsorption of amino acids onto bare ion oxides has primarily been attributed to interactions involving the carboxyl group,^36^ although hydrogen bonding and polar interactions involving oxygen and nitrogen atoms in the amide group may also contribute to the binding mechanism.

At high concentrations, the deviation of the values for the NaCl-containing samples increases significantly, likely due to uncertainties of the analytical method as well as the dilution and filtration required for the sample preparation. NADA concentrations were quantified by HPLC (see Experimental Section for details). The experimental protocol allowed reliable quantification up to 0.5 g L^−1^; therefore, samples with higher concentrations required dilution prior to analysis. The potential influence of sample filtration was analysed separately and resulted only in small deviations.

Both NADA isotherms lead to saturation partitioning values of approximately 1–10% on the nanoparticle surface. Hence, for every ten NADA molecules in the system, less than one of them is on the surface, corresponding to relatively low recovery. However, the partitioning strongly depends on the amount of nanoparticles present in suspension and could likely be increased by using higher nanoparticle concentrations. The presence of large amounts of amino acids on the surface can lead to a potential polymerization at the surface, phenomena that have been discussed in the context of the origin of life.^37^ Moreover, physisorption has been proposed as a possible precursor step to chemisorption under certain experimental conditions.^38^ High levels of biomolecule accumulation on surfaces have also been reported for montmorillonite layers, where large quantities of amino acids (about 200 g g^−1^), proteins, and lipids have been associated with the formation of oil reservoirs.^39,40^ These observations highlight the strong adsorption capacity of high surface area mineral materials and their potential applications that remain largely unexplored.

A notable feature of both NADA isotherms is the appearance of a small plateau at equilibrium concentrations of around 1–1.5 g L^−1^ NADA. This flattening of the curve might indicate a change in the adsorption mechanism on the nanoparticle surface.

The experimental isotherm data for both NADA systems were fitted using three widely applied isotherm models: Langmuir, Freundlich, and Redlich–Peterson. As shown in Fig. 1, none of the models provides a satisfactory description of the impure NADA, indicating that the adsorption mechanism is more complex than assumed by these simplified models. The isotherm exhibits distinct low- and high-concentration regimes, consistent with non-ideal adsorption behaviour and surface heterogeneity. Model equations, parameters and data points are presented in the SI (see Tables S2 to S5 and equations).

For the Freundlich model, the low affinity constant (*K*_f_ = 0.029) and the relatively poor coefficient of determination (*R*^2^ = 0.759) suggest that simple heterogeneous multilayer adsorption in the classical Freundlich does not adequately describe the system. The Langmuir model, which assumes homogeneous surface binding sites and monolayer coverage, yields a somewhat better fit (*R*^2^ = 0.861). Although the Langmuir constant (*k*_L_ ≈ 0.18 L g^−1^) and the dimensionless separation factor (*R*_L_ = 0.94) indicate thermodynamically favourable but weak adsorption, the calculated maximum capacity (*q*_max_ = 0.16 g g^−1^) exceeds the value expected for a NADA monolayer, suggesting multilayer adsorption and additional loading mechanisms.

The Redlich–Peterson model, which combines features of the Langmuir and Freundlich equations to account for non-ideal adsorption behaviour, shows intermediate agreement (*R*^2^ = 0.793). Its low isotherm constant (*K*_r_ = 0.035 L g^−1^) confirms the weak adsorption affinity indicated by the other two models, while the exponent *β* = 0.879 (<1) indicates predominantly Langmuir-like behaviour with contributions from surface heterogeneity. However, the experimentally observed capacities exceeding plausible monolayer coverage, together with the two regimes, support multilayer adsorption as a dominant contribution at higher loadings.

The limited performance of the classical models suggests that additional processes, such as nanoparticle aggregation or structural rearrangements of NADA, may influence the isotherm shape. Furthermore, the relatively large standard deviation in the impure NADA dataset limits the reliability of model fitting and reduces the justification for selecting a specific isotherm model.

In contrast, the pure NADA system yields higher overall correlation coefficients, with the Langmuir (*R*^2^ = 0.941) and particularly the Redlich–Peterson model (*R*^2^ = 0.964) providing better fits. The Langmuir capacity (*q*_max_ = 0.26 g g^−1^) is approximately 60% higher than for the impure system. However, the separation factor (*R*_L_ = 0.92) and the Langmuir constant (*k*_L_ ≈ 0.17 L g^−1^) still indicate weak adsorbent–adsorbate affinity, consistent with the low partitioning of NADA. The Redlich–Peterson constant *K*_r_ (0.028 L g^−1^) similarly reflects weak interaction. The large exponential parameter (*β* = 1.923) indicates a strong deviation from ideal Langmuir behaviour and shifts the model toward a Freundlich-like response, indicating that multilayer adsorption and surface heterogeneity dominate even in the purified system. Although the Freundlich model captures this heterogeneity (*R*^2^ = 0.777), it does not adequately describe the experimental data. Overall, the Redlich–Peterson model provides the best empirical description of the system, as it effectively integrates both the heterogeneous multilayer characteristics (Freundlich like behaviour, *β* > 1) and the fit to the experimental data (*R*^2^ = 0.964). While the model lacks direct physical interpretation, its parameters suggest that both Langmuir and Freundlich type contributions govern the adsorption equilibrium.

**Fig. 2:**
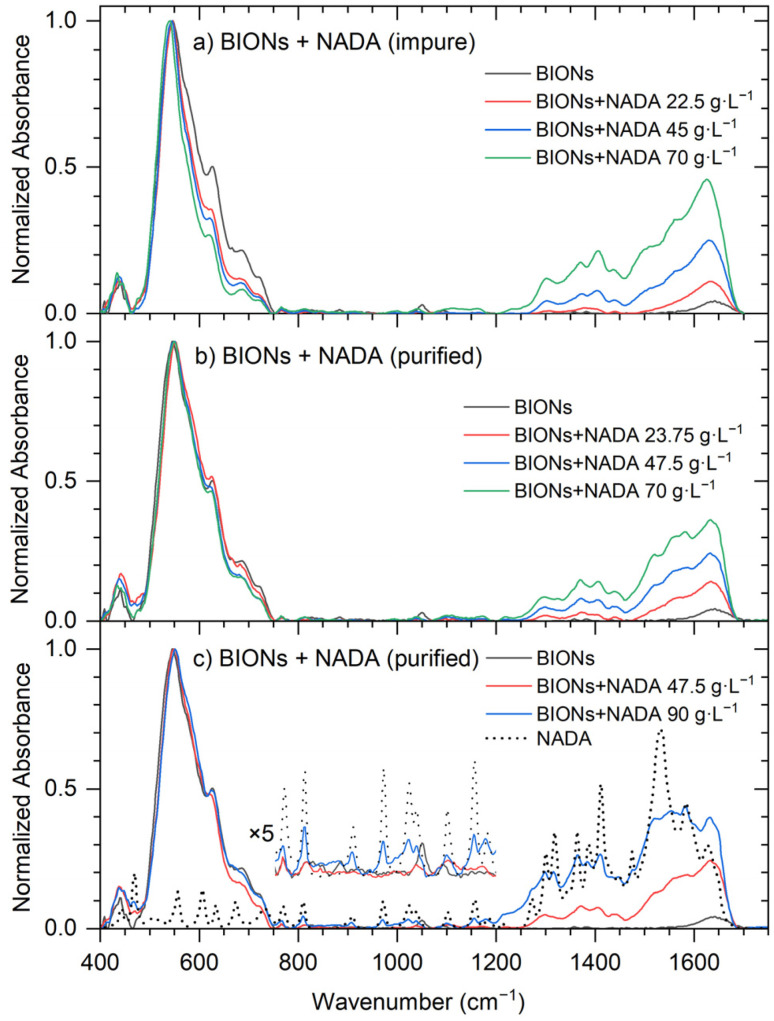
FTIR spectra of BIONs and BIONs after incubation with different concentrations of (a) impure NADA solution and (b) pure NADA solution. In (c) the spectra of crystalline NADA purified with ethanol is added for comparison.

Fig. 2 compares the FTIR spectra of BIONs and BIONs incubated with increasing concentrations of impure NADA (Fig. 2a) and purified NADA (Fig. 2b), recorded after washing and lyophilization. In both cases, the spectra are dominated by a strong absorption band at 550–650 cm^−1^, characteristic of Fe–O stretching vibrations, in agreement with previous reports.^41,42^ Upon incubation with NADA, additional absorption bands appear and increase systematically with concentration in the 1200–1700 cm^−1^ region. These bands are absent in the spectrum of pristine BIONs and are therefore assigned to adsorbed NADA molecules. They correspond to amide-related vibrations, amine functionalities, and C–N stretching.^43,44^ The broad features and increasing band intensities indicate a gradual increase in surface coverage with increasing NADA concentration.

At highest concentration (90 g L^−1^), the spectrum shows additional features that match those of the dried pure NADA reference (Fig. 2c). In particular, the region between 750 and 1200 cm^−1^, which remains largely featureless at lower concentrations (≤70 g L^−1^), develops several absorption bands corresponding to those of the crystalline NADA. This observation indicates that above a certain surface loading, NADA–NADA interactions become more important in addition to BION–NADA interactions. Such interactions may involve hydrogen bonding or ionic pairing between NADA functional groups within the adsorbed phase, indicating the formation of multilayer structures. Alternatively, clustering of NADA in solution followed by adsorption of these clusters onto the BIONs may contribute to this behaviour. Similar trends have previously been reported for other amino acids adsorbed on BIONs.^19^

The spectra in Fig. 2 confirm the presence of NADA adsorbed onto the BIONs for all tested concentrations and qualitatively show the gradual increase in amino acid loading with increasing solution concentration. Notably, while the dominant peak in the pure NADA spectrum appears around 1530 cm^−1^, the strongest peak in the solid phase spectra occurs near 1630 cm^−1^ and gradually shifts to 1626 cm^−1^ as the concentration increases. This shift may be attributed to enhanced hydrogen bonding in the amide group, potentially resulting from increased retained water or stronger intermolecular interaction between NADA molecules.^45^

The higher intensity of the 1630 cm^−1^ band compared with pure NADA may also reflect increased water retention within the NADA layer on the particle surface, which is plausible given the known osmoprotective properties of NADA.^46^ Such water retention could also arise from the formation of surface aggregates containing both NADA molecules and water acting as bridging species. Although the exact origin of the 1630 cm^−1^ band cannot be determined with certainty, the proportional increase of NADA-related signals is still evident at lower wavenumbers, including the band near 1400 cm^−1^ associated with asymmetric carboxylate vibrations. Slight differences in the peak profiles between the two NADA samples may further indicate the impact of salt ions.^45^

Light microscopy was used to investigate the effect of NADA adsorption on the distribution of BIONs. The images in Fig. S2 reveal increased agglomeration with rising NADA concentrations in solution and greater agglomeration than with bare BIONs. This behaviour was further confirmed by static light scattering (SLS), which showed a broad size distribution ranging from around 2 to nearly 90 µm in diameter (see Fig. S3), with *d*_50_ < 9 µm and *d*_90_ ≈ 17 µm. Increasing NADA loading on the BIONs surface resulted in a slight growth of smaller agglomerates and an apparent narrowing of the size distribution. However, the SLS size distribution differences are not substantial. Although SLS and light microscopy provide different perspectives on the system, both methods clearly indicate pronounced particle agglomeration, which is also observable for the BIONs before NADA coating.

### No NADA adsorption from a saline lysate

NADA is a highly soluble amino acid with compatible solute properties.^13,14^ We therefore investigated whether and to what extent it adsorbs onto BIONs in the presence of a complex mixture of molecules and ions. For this purpose, we used the lysate of the microalgae *M. salina*, a marine species with industrial potential^47^ and a system with which we have previously worked.^20,31,48,49^ Although it might have been more relevant to perform these experiments with *Pseudomonas elongata*, where NADA is produced as an intermediate in the ectoine biosynthesis pathway,^13^ preliminary experiments revealed severe analytical challenges due to the very high salt concentration. We therefore chose *M. salina* lysate as a complex system that could be analysed more reliably.

The adsorption experiments with impure NADA in the previous section showed that the presence of salt ions reduces NADA adsorption but does not fully inhibit it. The aim of the present experiments was therefore to evaluate how strongly adsorption is affected in a highly saline and biomolecule-rich environment. The microalgae lysate was supplemented with impure NADA at two different concentrations, 3 g L^−1^ and 0.75 g L^−1^, which corresponded to NADA-to-protein ratios of 1 g g^−1^ and 0.25 g g^−1^, respectively. NADA concentrations were quantified by HPLC after preliminary validation of the analytical method for the complex matrix. The corresponding protein adsorption data for different BION-to-protein ratios are presented in Fig. S4.

NADA concentrations in the supernatant of the adsorption experiments were quantified by HPLC. The measured concentrations in all samples (including controls without BION incubation and supernatants after magnetic separation) were virtually identical and closely matched the expected initial concentrations. These results indicate that no measurable NADA adsorption occurred during the incubation with the lysates. To confirm this observation, the analysis was repeated using a second chromatographic method. The first measurements were performed with a polyamine II column, while the second employed a LUNA HILIC Phenomenex column. Both methods consistently confirmed negligible NADA adsorption in presence of the microalgae lysate. This finding is notable because small molecules like NADA diffuse more rapidly in aqueous medium than larger biomolecules like proteins or polysaccharides and can adsorb in large quantities onto BIONs in simpler systems. However, in the complex lysate environment, larger biomolecules appear to displace the small, highly soluble amino acids from the nanoparticle surface, at least with the 1 h incubation time. Under the saline conditions of the microalgae lysate, proteins and fatty acids can potentially be separated using BIONs,^31^ while the highly soluble osmoprotective molecule NADA remains almost entirely in the supernatant with virtually no loss. This behaviour suggests that magnetic separation could serve as an effective first purification step for NADA by selectively removing other cellular components with minimal loss of the target molecule. Even if the permanence of NADA in the liquid phase was not the original expected outcome of these experiments, this result is still highly interesting for bioseparation purposes. Whether similar behaviour occurs for other amino acids remains to be investigated.

**Fig. 3:**
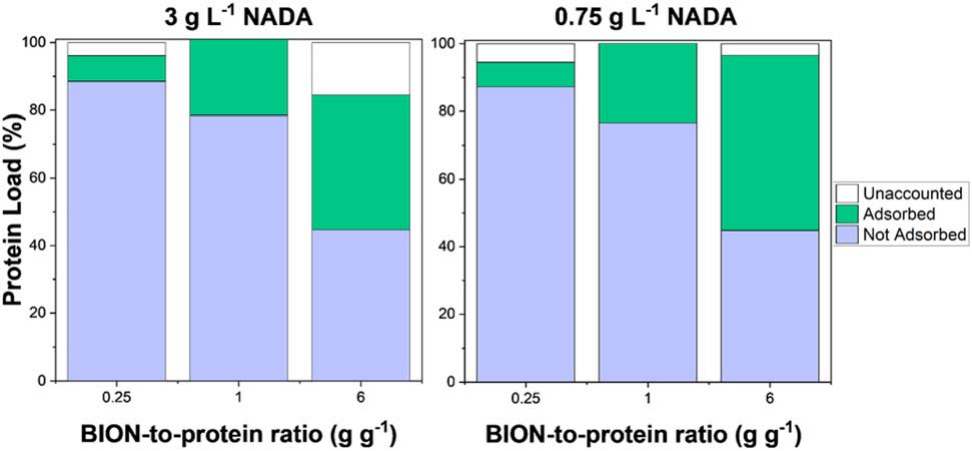
Protein adsorption onto BIONs with varying impure NADA concentration. Mass balance for proteins onto the BIONs from an *M. salina* lysate supplemented with 3 g L^−1^ NADA (left), and 0.75 g L^−1^ NADA (right).

After incubation and magnetic separation, a maximum of around 50% protein adsorption was observed, consistent with our previous experiments.^20,31^ Protein separation from cell extracts using magnetic nanoparticles has been reported using surface modification such as gold coatings^50^ or polymers layers,^51^ as well as amino acid homopolymer tags enabling selective interactions with BION surface^52^. Protein adsorption in our experiments was confirmed by BCA assays of the supernatants and independently validated by quantifying the protein fraction associated with the BIONs. The resulting protein mass balance after incubation for the three selected BION-to-protein ratios is shown in Fig. 3.

The FTIR spectra in Fig. S5 shows substantially greater complexity compared with the spectra discussed in the previous section, reflecting the presence of multiple biomolecular components in the lysate. The overall spectra profiles in both experiments are similar and resemble the magnetite spectrum in their relative intensities. Importantly, the absence of the characteristic NADA bands suggests that NADA adsorption onto the BIONs was negligible in both cases.

The spectra for the two NADA/protein ratios exhibit differences in the relative intensities of the major peaks as the BION-to-protein ratio changes (*e.g.*, in the region between 1465 cm^−1^ and 1650 cm^−1^ in Fig. S5). These variations may indicate changes in adsorption selectivity as the available nanoparticle surface area increases, which could influence the fractionation of different biomolecular components from the mixture, as previously observed.^20,53^ In addition, at lower BION ratios the absorbance across the main spectral regions increases proportionally, suggesting higher loading per particle even though the overall fraction of separated protein is smaller. This might indicate agglomeration of biomass. Lower absorbance at higher BION ratios indicates fewer adsorbed molecules per particle and thus greater direct interaction between the biomolecules and the BION surface. This observation supports the hypothesis that more proteins might be strongly bound under these conditions and therefore are not fully accounted for in the protein mass balance.

Although the complexity of the lysate limits precise molecular assignments, tentative interpretation of the most prominent bands is possible. The band at 850 cm^−1^ may correspond to conformational vibrations of DNA helices,^54^ although contributions from polysaccharides cannot be excluded.^55^ Bands at 1081 cm^−1^ likely arise from overlapping signals, including P–O symmetric stretching of nucleic acids, the amide III band of proteins, and C–O–H vibrations of carbohydrates. These bands are accompanied by another amide III component and the asymmetric stretching of P–O near 1235 cm^−1^.^56^ At 1467 cm^−1^, signals may originate from CH_2_ bending vibrations of lipids as well as the amide II band of proteins.^43^ Finally, the band at 1651 cm^−1^ corresponds to the amide I vibration of proteins, although contributions from carbonyl groups in nucleic acids may also be present.^56^ It should be noted that the bending vibration of water also appears in this region (around 1630 cm^−1^). Overall, the spectra are consistent with the presence of the main biomolecular classes found in living cells. Despite the relatively high concentrations of NADA in solution, no distinct NADA-specific bands could be identified in the spectrum.

### Phosphate adsorption as a monolayer

**Fig. 4:**
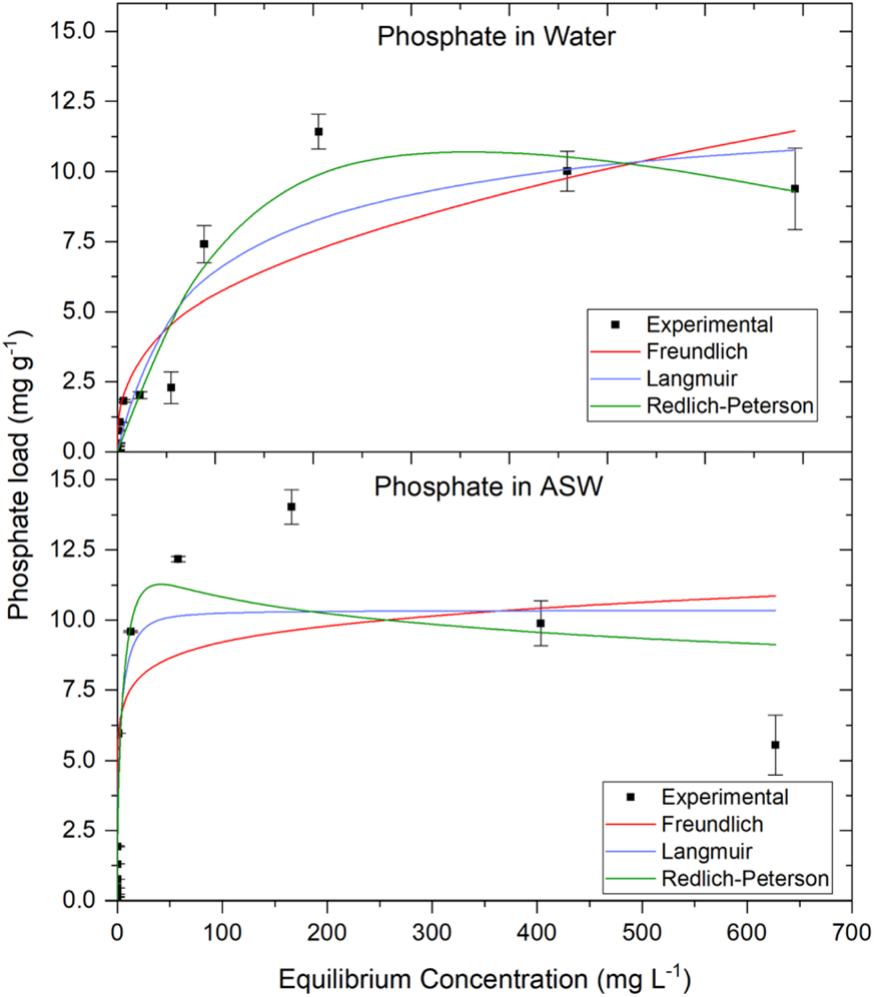
Adsorption isotherms of phosphate in dd-H_2_O (pH 7.07 and 7.42) and in ASW medium (pH 6.3–6.7). Data points originate from nine replicates: three independent experiments and analytical triplicates of each experiment (three independent samples out of each individual incubation experiment). Additionally, the fits based on the Langmuir, Freundlich and Redlich–Peterson models are presented.

In addition to NADA, we investigated the adsorption behaviour of a chemically distinct ion, phosphate, on BIONs. Phosphate is known to adsorb onto iron oxides^57^ and mineral surfaces in general *via* surface complexation mechanisms, including mono- and bidentate inner-sphere complexes as well as outer-sphere interactions mediated by the solvation shell.^58^ In some cases, precipitation of phosphate on mineral surfaces has also been reported.^58^ Most studies focus on phosphate removal from water due to its role as an environmental contaminant.^59^ However, phosphate is also of considerable biological and technological importance, as described in the Introduction. The resulting adsorption isotherm of phosphate on BIONs is shown in Fig. 4. The concentration range we investigated was shifted toward lower solution concentrations, compared to the NADA experiments, to take advantage of the high sensitivity and reproducibility of the molybdate-based quantification in the low-concentration regime. However, preliminary experiments revealed that deviations increased markedly with rising phosphate concentrations and successive dilution steps. To avoid the uncertainty observed for the NADA system, we decided to substantially reduce the concentration range.

Throughout this section, the abbreviation PO_4_ is used for all phosphate species. The maximum phosphate adsorption in water reached 11.43 mg L^−1^. Based on this saturation capacity, approximately 0.93 molecules nm^−2^ adsorb onto the surface at equilibrium, assuming a radius of 0.38 nm for phosphate in aqueous solution,^60^ corresponding to a projected area of about 0.45 nm^2^, which represents roughly half of the required for a complete monolayer. In the literature, PO_4_ adsorption on mineral surfaces is typically described as monolayer adsorption unless positive ions or water act as intermediates enabling further adsorption.^57^ Interestingly, the isotherm in water shows a small plateau at equilibrium concentrations between 6 and 55 g L^−1^, similar to the behaviour observed for NADA. This may indicate an early saturation of specific bonding sites, followed by adsorption through alternative mechanisms, such as different binding modes or adsorption of small aggregates. However, this interpretation remains speculative. A comparable phenomenon has been reported for protein coronas, where an early-stage surface saturation can occur at surface densities well below monolayer coverage.^61^ A similar effect may also contribute to the behaviour observed for phosphate in this system.

The adsorption capacities obtained here are comparable to values reported for similar particles at comparable pH conditions.^57,62–64^ While Ajmal *et al.*^57^ report 58 mg g^−1^ at pH 7 for a magnetite with higher specific surface area than ours, Lou *et al.*^62^ report values below 6 mg g^−1^ at pH 7 for a magnetite also with a higher specific surface area than ours. Huang^63^ reports approx. 1.5 µmol m^−2^ for a hematite and Yoon *et al.*^64^ report 3 mg_P_ g^−1^. Phosphate adsorption on iron oxides is generally attributed mainly to electrostatic interactions, although adsorption capacity can vary significantly with pH and ionic strength, making direct comparison between studies difficult.^63^ Phosphate adsorption has also been widely reported for many natural and synthetic materials.^65^ Materials with very high surface areas, such as metal–organic frameworks (MOFs), can exhibit particularly high phosphate adsorption capacities.^65^ It remains unclear to what extent the adsorption capacity of BIONs could increase under more favourable conditions, such as lower pH or higher phosphate concentrations approaching the solubility limits. Nevertheless, to our knowledge, reports of phosphate multilayer formation on surfaces are not available.

In aqueous environments with high ionic strength, electrostatic forces are strongly shielded. While such shielding could potentially increase phosphate adsorption, high salt concentrations also promote nanoparticle agglomeration and may reduce phosphate solubility. To investigate this effect, we examined phosphate adsorption in artificial seawater (ASW). While for NADA we were strongly interested in discovering how NADA would adsorb in competition with a lysate, for phosphate we already knew that it would deplete proteins from the surface,^23,24^ so the focus shifted to studying how high ionic strength (the highly saline environment of ASW) would affect the phosphate interaction with the surface. As shown in Fig. 4, adsorption increases more steeply with PO_4_ concentration than in pure water. We attribute this behaviour to the reduced solubility of phosphate at high ionic strength.^48^ Consistent with our previous observations for amino acids,^19^ lower solubility in solution appears to favour partitioning to the solid. The slope of the adsorption isotherm, however, also depends on additional parameters, including nanoparticles-to-solute ratio and the charge density of both the solute and the particle surface, which were not varied in the present study.

At phosphate concentrations higher than 250 mg L^−1^, a strong decrease in adsorption is observed. Similar reductions in loading capacity have previously been observed for several (bio)molecules on BIONs, including small molecules such as the fatty acid salt oleate,^66^ peptides,^35,52^ and proteins.^20^ In earlier studies, this behaviour was tentatively attributed to analytical uncertainties arising from the multiple dilution steps required for quantification at high solute concentrations. However, in the present case analytical limitations can be excluded, as the calibration range for PO_4_ extends well beyond the highest concentrations used in the isotherm measurements. We therefore hypothesize that increasing PO_4_ concentration, which influences the overall chemical potential of the system,^67^ leads to an inflection point in the adsorption isotherm. This behaviour may arise from processes such as increased nanoparticle agglomeration or condensation phenomena in solution, both of which could reduce the effective loading of PO_4_ onto the BION surface. Precipitation of phosphates on mineral surfaces has frequently been reported in the literature and is often closely associated with adsorption processes.^68–70^ In aqueous systems, PO_4_ is also known to form precipitates readily, particularly in the presence of di- and trivalent metal ions.^29,48^ Similar condensation or structural reorganisation may also contribute to the unexpected decrease in adsorption observed previously for proteins^20,31^ and lipids^31^ in lysates, although this remains an open question.

The experimental PO_4_ adsorption data in water were fitted using the Freundlich, Langmuir, and Redlich–Peterson isotherm models, following the same approach used for NADA (see Tables S6 and S7 for data points and parameters of the models, respectively).

For adsorption in water, the Freundlich model yields a *K*_f_ value of 1.098, indicating moderate adsorption capacity, although the relatively low coefficient of determination (*R*^2^ = 0.852) reveals limited predictive capacity. The Langmuir model provides a better fit (*R*^2^ = 0.909) and predicts a maximum monolayer capacity of 12.0 mg g^−1^, supporting the plausibility of monolayer adsorption. The Redlich–Peterson model shows the best agreement with the experimental data (*R*^2^ = 0.956). Its constant (*K*_r_ = 0.094 L mg^−1^) indicates moderate adsorption while the exponent *β* = 1.692 (*β* > 1) suggests behaviour approaching Langmuir-type adsorption but with additional complexity.

For adsorption in ASW, the Freundlich model again provides the poorest description (*R*^2^ = 0.743), although the higher *K*_f_ value (*K*_f_ = 5.414) indicates favourable adsorption. The Langmuir model predicts a maximum monolayer capacity of 10.3 mg g^−1^ and improves the fit (*R*^2^ = 0.847). The Redlich–Peterson model yields a comparable fit (*R*^2^ = 0.833), with a high *K*_r_ (8.07 L mg^−1^) and an exponent *β* = 1.090 close to unity, suggesting adsorption behaviour approaching Langmuir-type monolayer adsorption on a relatively homogeneous surface. Data points and model parameters are found in Tables S8 and S9, respectively.

In sum, phosphate in ASW shows a steeper slope at lower concentrations than in water, as reflected in a higher Langmuir-type *k*_L_ value (see Tables S7 and S9) and therefore an apparently higher affinity for the surface. We attribute this behaviour partly to thermodynamic effects, as the reduced solubility of phosphate ions in ASW promotes partitioning to the solid phase, and partly to kinetic effects resulting from electrostatic shielding.

Overall, none of the classical models provide a fully satisfactory description of the experimental data, which indicates that the adsorption mechanism is more complex than assumed by these simplified models. Incorporating additional factors such as solute–solute interactions or particle aggregation effects into adsorption models may improve their predictive capability.^71^ Comparable deviations from standard isotherm models have only rarely been reported in the literature.^72,73^

**Fig. 5:**
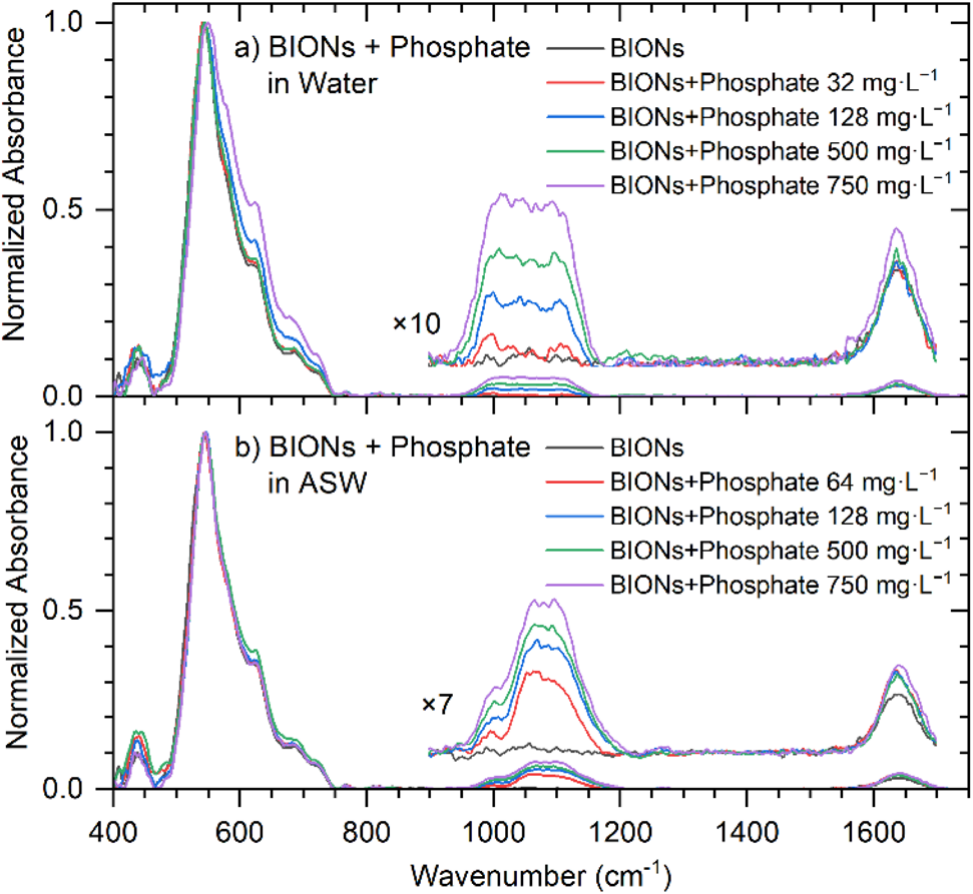
FTIR spectra of BIONs and BIONs after incubation with different concentrations of phosphate (a) in ddH_2_0, and (b) in ASW medium.

Fig. 5 shows the FTIR spectra of pure BIONs and BIONs exposed to phosphate at different concentrations in two solvents. Panel (a) presents spectra measured in deionized water (dd-H_2_O), while panel (b) shows spectra measured in ASW. In both cases, the spectra reveal similar features in the range of 400–1750 cm^−1^. For phosphate-treated BIONs in dd-H_2_O (Fig. 5a), a strong absorption band at 550–650 cm^−1^ is observed, characteristic of Fe–O vibrations.^41,42^ In addition, a weak and broad band appears between 950 and 1150 cm^−1^, whose intensity increases with increasing phosphate concentration, and which is absent in the spectrum of pure BIONs. These features are attributed to P–O stretching modes. The strong spectral broadening indicates heterogeneous surface coordination rather than the presence of free phosphate species. Similar spectral features have been reported for phosphatated iron-containing minerals such as hematite, goethite, maghemite, and magnetite.^74–78^ Absorption in this region is commonly associated with the formation of inner-sphere Fe–O–P surface complexes on iron oxide nanoparticles.^75,78,79^

For comparison, Fig. 5b shows the spectra of phosphate-treated BIONs in ASW. The strong band at 540–650 cm^−1^, as well as the features below 500 cm^−1^, remain essentially unchanged compared with the samples measured in dd-H_2_O. The phosphate-related bands appear at the same spectral positions, although the inset of Fig. 5b reveals a slightly different band shape and lower intensity of the absorption feature at ∼1000 cm^−1^. This decrease may indicate reduced phosphate adsorption in ASW. Such behaviour can be explained by competitive adsorption effects, as ions such as sulphate may compete with phosphate for surface adsorption sites.^80^ In addition, dissolved salts increase the ionic strength of the solution and can influence both phosphate adsorption and the formation of Fe–O–P surface complexes.^75,78^

The broad band between 950 and 1150 cm^−1^ likely corresponds to overlapping contributions from several phosphate related vibrations, *e.g.*, the P=O stretching (around 1100 cm^−1^), P–O and P–OH vibrations (1000–1100 cm^−1^) and P–OFe bond (around 1020 cm^−1^).^57,64,74,81^ The band broadening suggests the coexistence of multiple adsorption complexes, possibly including mono and bidentate complexes in both protonated and deprotonated forms. At higher pH than those investigated here, Daou *et al.*^74^ reported that mononuclear protonated complexes may dominate. However, some of the spectral features observed in ASW may also correspond to outer-sphere complexes, consistent with the similarity between the bands and those of free phosphate.^45^

**Fig. 6:**
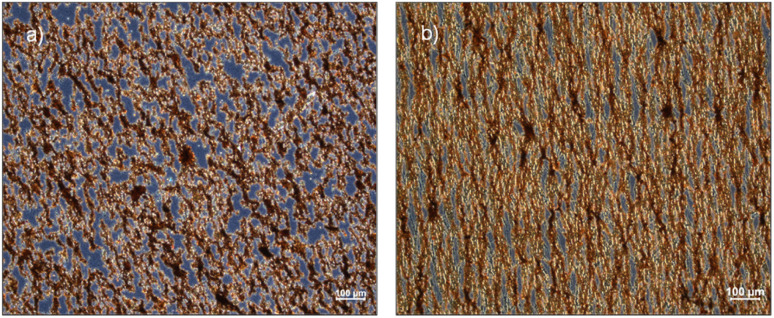
Phase contrast images of BIONs contacted with different phosphate concentrations in dd-H_2_O (10× magnification, 10 g_BION_ L^−1^). (a) BIONs incubated with 128 mg_PO_4__ L^−1^. (b) BIONs incubated with 750 mg_PO_4__ L^−1^.

To further investigate particle behaviour, light microscopy was used to examine the agglomeration of phosphate loaded BIONs. In phase contrast mode, we observed that the phosphate layer influences particle interactions at higher loadings in dd-H_2_O (see Fig. 6 and S6 for BIONs without PO_4_ and for the lowest PO_4_ loading). This effect was not observed for samples incubated in ASW.

Fig. 6b (phosphate concentration of 750 mg L^−1^) shows a noticeable alignment of the particles along the field lines. A similar effect was observed in our previous experiments,^49^ although those systems involved significantly larger particles and lower phosphate concentrations. We suspect that the 10× objective of the light microscope generates a weak magnetic field that induces particle alignment. However, the effect appears to be influenced by the presence of phosphate on the particle surface. Some studies report a slight decrease in magnetization of magnetite when phosphate adsorbs onto its surface.^81^ To our knowledge, however, no previous reports describe the type of particle alignment observed here. Phosphorous atoms have been reported to modify the magnetic properties of iron-containing compounds,^82,83^ suggesting that phosphate complexation at the BION surface may influence the magnetic response of the particles. This effect could occur either directly through modification of the surface magnetic properties or indirectly through changes in particle agglomeration and the resulting magnetic interactions. The hydration layers associated with both phosphate ions and the nanoparticles may also contribute to the observed magnetic ordering.

Static light scattering (SLS) measurements revealed large agglomerates in the micrometre range (Fig. S7), like those observed for NADA. Although the strong negative charge of phosphate might be expected to promote particle dispersion,^64^ the SLS data do not support this assumption. Instead, samples incubated in both water and ASW showed similar particle size distributions (Fig. S8). Interestingly, particle alignment in the weak magnetic field of the microscope was only observed for samples incubated in water and not for those incubated in ASW (Fig. S9).

The coexistence of adsorption and precipitation processes during the incubation of BIONs with phosphate in multicomponent ionic solutions raises additional questions. According to Yalin *et al.*,^70^ adsorption should be preferential compared to precipitation at the pH range and low concentrations used in our experiments. However, as PO_4_ concentrations increase in the presence of multiple cations, the formation of phosphorus-containing precipitates becomes more likely. Under such conditions, nucleation in solution may reduce the number of phosphate ions available for adsorption onto the nanoparticle surface. In addition, the increased ionic strength of the system may enhance BION agglomeration, which could impose diffusional limitation on PO_4_ transport to the nanoparticle surface and thereby reduce the phosphate-to-BION loading ratio.

### Capacity increase upon consecutive incubation

After studying the adsorption of the two target molecules individually, we performed sequential experiments to determine whether the loading behaviour depends on the molecule initially forming the corona on the BION surface. Moreover, we wanted to establish if phosphate would elute NADA from the surface (or if NADA would elute phosphate). For this purpose, NADA and phosphate coated particles were prepared separately. After two washing steps (each incubated 5 min in water), the adsorption of each molecule onto the pre-formed layer of the other one was investigated.

**Fig. 7:**
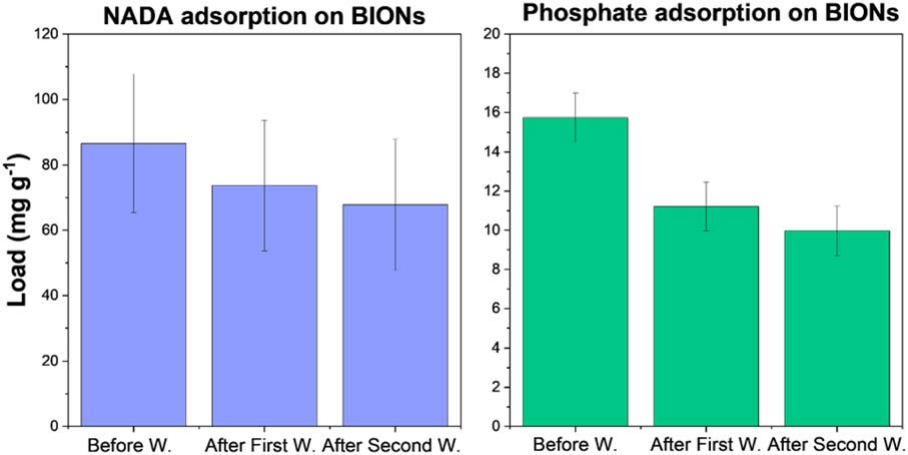
NADA (left), and phosphate (right) adsorption capacities on BIONs after 1 h incubation and two short and vigorous washing steps (W.) with dd-H_2_O water. In both cases the adsorbed layer should occupy most of the BION surface.

BIONs were first incubated with 12.5 g L^−1^ NADA solution, resulting in NADA-coated particles with a loading of approximately 86.6 mg_NADA_ g_BION_^−1^ (Fig. 7a). Subsequent washing caused partial desorption of NADA: about 15% of the adsorbed layer was removed during the first wash and an additional 7% during the second wash, resulting in a final coating of approximately 68 mg_NADA_ g_BION_^−1^. The washing steps were performed under vigorous mixing (1000 rpm) to remove weakly adsorbed molecules though convective effects.

Assuming a homogeneous distribution of NADA on the surface and an approximate molecular diameter of 0.5 nm,^19,84^ 68 mg_NADA_ g_BION_^−1^ corresponds to roughly three NADA molecules nm^−2^ remaining on the particle surface after washing. This capacity is consistent with complete monolayer coverage or slightly higher for quite tightly packed molecules. However, such calculations are largely fictive and should render only a notion of possible dimensions. Another approach might be to imagine the NADA molecules less densely packed and employ a lower surface occupancy as an orientation value, *e.g.* the “residue solvent accessibility”.^85^ In that approach the number of layers would be much higher, about six times more for the 68 mg_NADA_ g_BION_^−1^. Based on its molecular mass and its chemical groups, NADA might occupy a “residue solvent accessibility” area slightly below the value for the bulky phenylalanine and therefore close to the value of leucine, *i.e.* ∼2 nm^2^ for one molecule.^85^

The same procedure was applied for phosphate using a solution containing approximately 500 mg L^−1^ PO_4_. This resulted in an initial coating of about 15.74 mg_phosphate_ g_BION_^−1^. After two washing steps, 29% and 8% of the adsorbed phosphate were removed, respectively, yielding a final surface loading of approximately 10 mg_phosphate_ g_BION_^−1^ (Fig. 7b). The significant desorption observed during washing suggests that phosphate adsorption occurs through multiple mechanisms, as often reported in literature,^86^ including strong inner-sphere complex formation with the surface as well as weaker outer-sphere complexation mediated by the solvation shell. We assume that the outer sphere complexes reequilibrate rapidly and therefore desorb more readily when the particles are incubated in solute free solutions.

**Fig. 8:**
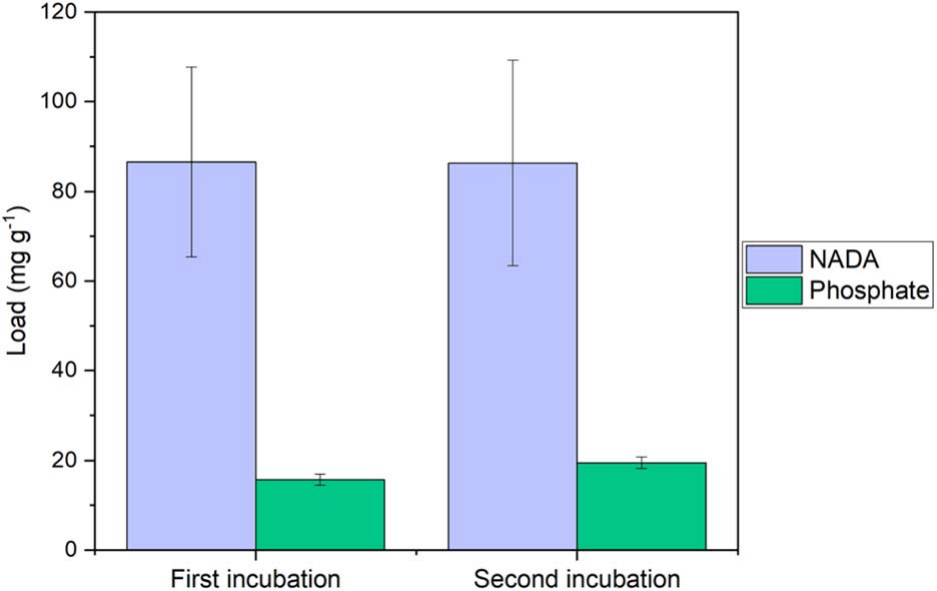
Comparison of the adsorption levels of NADA and PO_4_ on BIONs (left bars) and upon sequential adsorption onto previously loaded BIONs (right bars). Sequential adsorption of NADA onto BIONS loaded with ∼10 mg g^−1^ of phosphate, or of PO_4_ on BIONs loaded with ∼68 mg g^−1^ of NADA.

Sequential adsorption experiments were then performed by incubating phosphate coated BIONs with NADA (PO_4_-BION) and NADA coated BIONs with phosphate (NADA-BION). In both cases, the resulting adsorption capacities were comparable to those obtained for direct adsorption onto bare BIONs (Fig. 8a and b). The amount of phosphate adsorbed onto NADA-BION particles was approximately 24% higher than the adsorption observed prior to NADA coating. These results demonstrate that the BION surface can be readily modified through simple incubation steps, highlighting the potential of BIONs as versatile and low-cost carriers for separation applications.

**Fig. 9:**
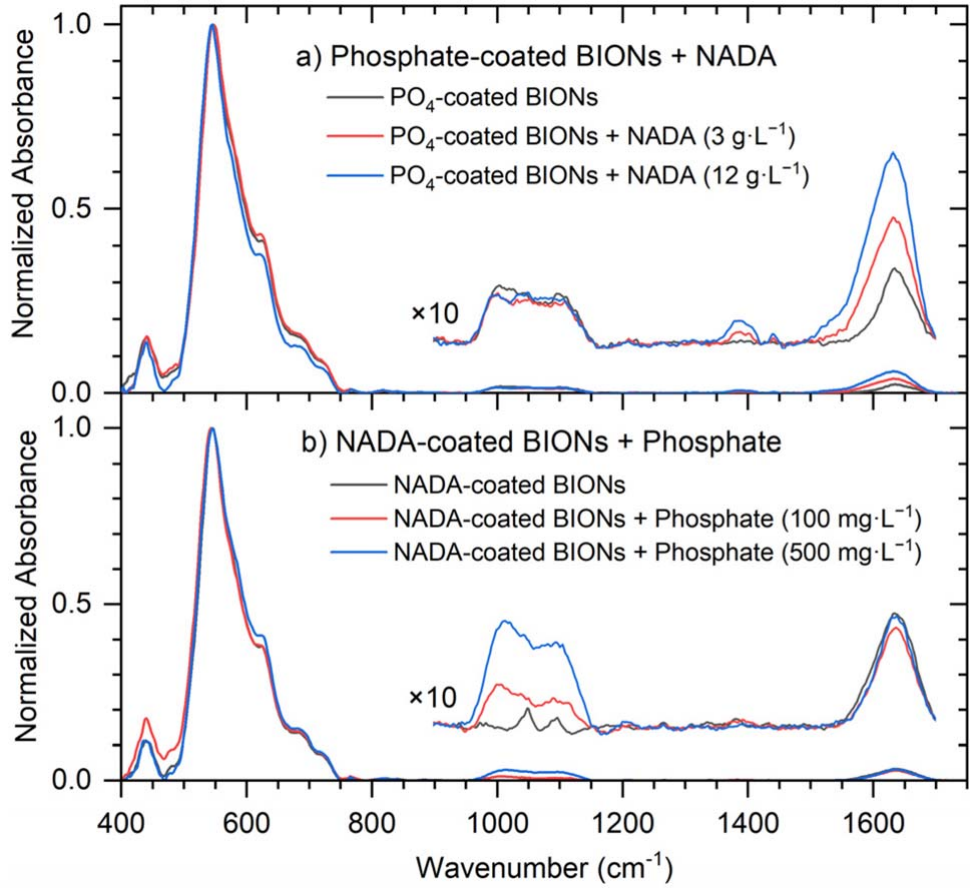
FTIR spectra of (a) phosphate-coated BIONs (PO_4_-BION) and PO_4_-BIONs after incubation with different concentrations of NADA in solution. (b) NADA-coated BIONs (NADA-BION) and NADA-BIONs after incubation with different concentrations of phosphate in solution.

The simultaneous presence of both components in the final corona was confirmed qualitatively through FTIR measurements (Fig. 9). Fig. 9a shows spectra of BIONs initially incubated with PO_4_ in aqueous solution and subsequently exposed to NADA. After phosphate adsorption, a broad absorption band appears in the region of 950–1150 cm^−1^, characteristic of adsorbed phosphates (see also Fig. 5). This band is attributed to P–O stretching modes associated with inner-sphere phosphate complexes on iron oxide surfaces. Upon subsequent adsorption of NADA, the phosphate-related band remains clearly detectable with no significant loss in intensity, indicating that the pre-adsorbed phosphate layer has not been removed. At the same time, NADA-related absorption bands around 1390 cm^−1^, 1440 cm^−1^, and 1500–1700 cm^−1^ increase systematically with increasing NADA concentration. The persistence of the phosphate signal together with differences in the spectral shape of the NADA-BION bands, compared to the shape in Fig. 2b and c, suggests that NADA forms an additional layer rather than displacing the phosphate from the iron oxide surface. A band near 1630 cm^−1^ is also observed, which is likely associated with adsorbed water, as the H–O–H bending vibration occurs in this region.^45^ Interestingly, the signal of this band still increases proportionally with NADA concentration, even though the initial NADA concentrations in these experiments were relatively low. A similar proportional increase is observed for weaker features around 1400 cm^−1^, further supporting the presence of NADA on the particle surface in addition to the phosphate layer.

Fig. 9b shows the inverse adsorption sequence, where NADA is adsorbed first, followed by phosphate exposure. After phosphate incubation, a pronounced absorption band appears in the 950–1150 cm^−1^ region, indicating substantial phosphate uptake despite the prior NADA coating. The presence of this band demonstrates that phosphate can still interact effectively with the system, even when the particle surface is pre-coated with NADA. Upon exposure to the second adsorbate, bands associated with the newly introduced species increase in intensity, while spectral features attributed to the pre-adsorbed NADA layer remain largely unchanged. The observed spectral changes are not systematic, suggesting that no clear displacement of the initial NADA layer can be identified in the FTIR data. The similarity of the phosphate band to that observed for phosphate adsorption on bare BIONs (Fig. 5a) further suggests that NADA does not fully block access to the iron oxide surface. Although FTIR alone cannot determine whether phosphate penetrates through the NADA layer or partially displaces NADA locally, the data indicate that phosphate adsorption is not restricted to interactions with the outermost organic layer. Similar behaviour has been reported for cobalt nanoparticles in presence of amino acids.^87^

### Ferromagnetic phosphate-BION response

Magnetic resonance methods are increasingly used to investigate magnetic interactions in biological and chemical systems. The magnetic properties of nanomaterials depend on factors such as composition, crystal structure, particle size, and interactions with the surrounding environment or surface coatings. Electron paramagnetic resonance (EPR) is particularly suitable for studying the structural and magnetic properties of paramagnetic, superparamagnetic, and ferromagnetic nanomaterials. To investigate the magnetic alignment observed in Fig. 6c and d, EPR measurements were performed on phosphate-loaded BION samples prepared in dd-H2O. For these experiments, a different batch of BIONs was used, synthesized and characterised as described previously,^27^ with properties comparable to those of the particles used in the experiments above. The nanoparticles were incubated with three phosphate concentrations (32 g L^−1^, 750 mg L^−1^ and 32 mg L^−1^) prior to the EPR measurements. These concentrations were selected to enable monitoring changes in the EPR characteristics across substantially different adsorption levels (the saturation value and significantly above and below).

**Fig. 10:**
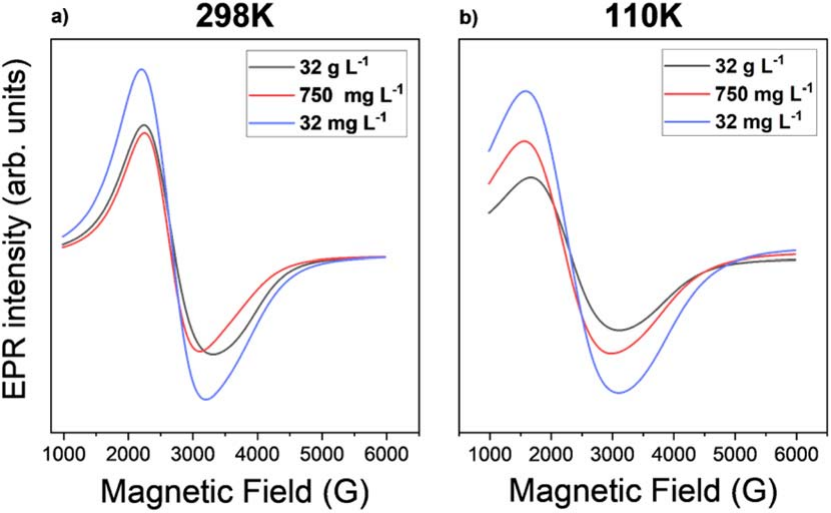
FMR spectra of the PO_4_-BION samples for three phosphate concentrations in solution at (a) 298 K and at (b) 110 K.

EPR spectra recorded at room temperature (298 K) for all PO_4_-BION samples exhibit a broad resonance line in the *g*-factor region of 2.6, with a spin concentration of about 10^20^ spin mm^3^ (Fig. 10a and Table 1). For ferromagnetic particles larger than 5 nm, strong exchange interactions lead to ferromagnetic resonance (FRM) signals whose intensity can exceed paramagnetic resonance signals by three orders of magnitude.^88,89^ The spectra of the magnetite-phosphate samples show broad resonance lines peak-to-peak width (Δ*H*) between 860 and 1060 G. This broadening arises from strong collective exchange interactions between the magnetic moments of Fe^3+^ ions, which generate molecular exchange fields and contribute to long-range magnetic ordering.^90^

**Table 1:** FMR spectral characteristics of the PO_4_-BION samples for three phosphate concentrations in solution at 298 and at 110 K

Sample	298 K		110 K	
	*g*-factor	Δ*H*, *G*	*g*-factor	Δ*H*, *G*
32 g L^−1^	2.59	1060	2.88	1440
750 mg L^−1^	2.62	1000	2.94	1515
32 mg L^−1^	2.65	860	3.03	1420

Magnetite nanoparticles possess an inverse cubic spinel structure, where Fe^2+^ and Fe^3+^ ions occupy tetrahedral and octahedral sites.^91,92^ The observed asymmetric resonance lines, with a symmetry parameter (A/B) between 1.2 and 1.4, are consistent with cubic magnetocrystalline anisotropy.^93,94^ The *g*-factor value near 2.6 (298 K) reflects the collective interaction of Fe^3+^ magnetic moments, while the broad absorption line is characteristic of the convoluted cubic crystal powder pattern of an FMR of fine-grained precipitates of single ferro- or ferrimagnetic domains.^95^

Upon cooling the magnetite–phosphate samples to 110 K, the signal amplitude decreases monotonically and the resonance lines broaden to approximately 1500 G, while the *g*-factor increases to 3.0,^95–98^ as shown in Table 1 and Fig. 10b. This behaviour is typical for FMR of single-domain particles, whose sizes do not exceed 30 nm, in the absence of transitions to a magnetically ordered state (spin-glass, “stable state”).^99,100^ The decrease in the external resonant field (the amplitude) at lower temperatures is explained by an increase in temperature-induced internal microscopic fields, such as the exchange anisotropy field. Exchange anisotropy arises from a disordered spin profile caused by antiferromagnetic interactions between magnetic clusters or neighbouring spins in Fe_3_O_4_ nanoparticles. It is well known that the resonance field, line width and intensity for coated and uncoated Fe_3_O_4_ nanoparticles strongly depend on temperature; the line broadens and shifts towards lower fields with decreasing temperature.^101^

Surface coatings can significantly influence the magnetic properties of magnetite nanoparticles, for example, by preventing aggregation, reducing surface oxidation, and modifying interparticle interactions. In our samples, the phosphate coating seems to act as a stabilizing layer that preserves the magnetic core and its integrity. All PO_4_-BION samples exhibit similar FMR spectral patterns. However, as the phosphate concentration in solution decreases from 32 g L^−1^ to 32 mg L^−1^, the spatial separation between nanoparticles likely decreases and the surface shielding becomes weaker. This behaviour is reflected in a shift of the *g*-factor toward lower magnetic fields, from 2.59 to 2.65, indicating stronger magnetic interactions.^102^ We interpret this effect as evidence of increased nanoparticle agglomeration or clustering.

Such changes in the FMR spectra are likely related to a reduction in the thickness of the isolating phosphate layer and to structural changes of surface defects caused by adsorbed phosphate molecules. As a result, dipole–dipole interaction between aggregated particles increases, leading to a shift of the broad resonance line toward lower magnetic fields.^93,103^ In addition, the narrowing of the FMR line from 1060 to 860 G with decreasing phosphate concentration (at 298 K) may reflect stronger exchange interactions, a phenomenon frequently observed in magnetic nanoparticles.^104^

Bianchetti and Di Valentin^105^ reported that coating magnetic nanoparticles with organic acids can enhance their saturation magnetization by reducing spin-flipping at octahedral Fe^3+^ sites and promoting additional ferromagnetic superexchange interactions.^105^ Although phosphate is not an organic acid, a similar mechanism may help explain the relatively high *g*-factor values observed in our PO_4_-BION samples and the pronounced magnetic alignment observed in the microscopy images (Fig. 6c and d). A dense phosphate coating may reduce nanoparticle aggregation and modify surface spin interactions, thereby enhancing the magnetic response of the particles.

In sum, the ferromagnetic resonance measurements in water suggest that the PO_4_ layer shields the BION surface, reduces agglomeration, and enhances the magnetic response of the particles, which increases with phosphate concentration. In contrast, no such magnetic response was observed in ASW, consistent with the microscopy observations. Whether nanoparticle agglomeration plays a central role in the apparent adsorption of large quantities of certain molecules on BIONS remains an open question.

## Conclusions

In this work, we studied the adsorption behaviour of two highly soluble small molecules: an organic amino acid derivative (NADA) and an inorganic anion (phosphate). NADA is an osmolyte capable of binding large amounts of water, while phosphate readily forms precipitates in the presence of various ions. Our objective was to obtain fundamental insights into their adsorption behaviour, including under saline conditions, and to explore how the resulting corona influences sequential adsorption.

The adsorption isotherms revealed that the saturation loading of NADA on BIONs clearly exceeds the capacity expected for a monolayer. At very high NADA concentrations, FTIR spectra show bands corresponding to crystalline NADA, which indicates the presence of NADA–NADA interactions with adsorbed layers. In addition, the IR data suggest the coexistence of inner- and outer-sphere complexes, consistent with both direct surface binding and interactions mediated through solvation shells.^106^ The high NADA capacities observed at saturation are comparable to those previously reported for proteins^20,23,27,31,107^ and other highly soluble amino acids.^19^ The appearance of spectral features resembling a crystalline structure on the nanoparticle surface suggests potential applications of BIONs in processes such as biocondensation and crystallization. Moreover, the observation that such small molecules can form adsorbed layers approaching the capacity observed for large biomolecules raises the question of what ultimately limits their growth.

For PO_4_, maximum adsorption capacities of approximately 12 and 14 mg g^−1^ were obtained in water and artificial seawater (ASW), respectively. These similar capacities across environments with very different ionic strengths demonstrate that BION-based separation remains effective under both low- and high-salinity conditions, despite the significant agglomeration observed for the nanoparticles.

FTIR spectra of phosphate adsorbed in water indicate direct P–OFe interaction associated with inner-sphere complexation,^75,78,79^ while the signals in ASW more closely resemble those of free phosphate, suggesting a larger contribution of outer-sphere interactions. This observation is somewhat counterintuitive, as increased ionic strength and electrostatic shielding would typically favour inner-sphere complexation due to competition for the outer-sphere.^57,108,109^ However, ASW contains several cations capable of forming phosphate precipitates, and the high salinity promotes nanoparticle agglomeration, which may influence the adsorption environment.

In sequential adsorption experiments, similar amounts of NADA adsorbed onto PO_4_-BIONs as onto bare BIONs. Additionally, phosphate showed comparable adsorption, even slightly higher (∼24% more), onto NADA-BIONs than onto bare BIONs. The presence of inner-sphere phosphate complexes on the NADA-coated particles indicates that the NADA corona does not fully block PO_4_ from interacting directly with the BION surface. Likewise, phosphate inner-sphere complexes remain detectable after NADA adsorption onto PO_4_-BIONs, *i.e.* NADA does not displace the adsorbed phosphate from the BION surface.

Another notable observation is that NADA did not adsorb onto BIONs in the presence of *M. salina* lysate (in ASW medium), most likely because other cellular components preferentially adsorb onto the nanoparticle surface. This behaviour suggests that BIONs could serve as an effective first purification step for NADA or similar osmolytes, yielding a supernatant with a significantly higher purity for NADA, after magnetic separation, than in the initial suspension.

Overall, our results highlight the largely untapped potential of bare iron oxides for separation applications, particularly in bioseparations and environmental processes such as wastewater treatment.^51^ For example, Kim *et al.* demonstrated that chitosan-coated magnetic nanoparticles can repeatedly remove phosphate from waste streams in flow-through systems.^110^ Our sequential experiments further show that solid–liquid partitioning is not governed solely by surface chemistry but also by additional factors as solution composition, particle aggregation, and competitive adsorption processes. A comprehensive understanding of these systems therefore requires consideration of binding site availability and mass transport effects, initial conditions (*e.g.*, pH and ionic strength), and the physicochemical properties of both, surfaces and adsorbates, as well as further studies on simultaneous and sequential adsorption of biomolecules onto nanoparticles.^21,22^

Our IR results also suggest that multiple adsorption mechanisms coexist in aqueous systems, including both inner-sphere and outer-sphere complexation pathways.^58,111^ As noted by Wang *et al.* in their work on sulphate adsorption on hematite:^80^ “The tendency to form both inner- and outer-sphere complexes on diverse minerals suggests that the types of sulfate complexes are controlled by the physiochemical properties of sulfate, likely the relative magnitude of the sulfate hydration and binding energies, involved in sulfate adsorption, more than the properties of mineral surfaces.” Future research on solid–liquid partitioning should therefore focus on linking solution phase interactions with adsorption processes occurring at and near solid surfaces.

## Author contributions

P. F.-G. conceptualization, data curation, formal analysis, investigation, methodology, project administration, resources, supervision, validation, visualization, writing – original draft, writing – review and editing; C. E. D.-C. data curation, formal analysis, investigation, methodology, validation, visualization, writing – original draft, writing – review and editing; S. S. K. data curation, formal analysis, investigation, methodology, visualization, writing – original draft, writing – review and editing; V. J.-D. formal analysis, investigation, methodology, visualization, writing – original draft, writing – review and editing; L. A.-C., methodology, resources, writing – review and editing; J. L., data curation, formal analysis, investigation, methodology, validation, visualization, writing – original draft, writing – review and editing.

## Conflicts of interest

There are no conflicts to declare.

## Supplementary Material

NA-OLF-D6NA00243A-s001

## Data Availability

Data for this article, including raw IR and EPR spectroscopy data, original microscopy files, and raw and processed UV-Vis data from plate reader and HPLC measurements are available at [https://doi.org/10.14459/2026mp1841328]. Further data supporting this article have been included as part of the supplementary information (SI). Supplementary information: adsorption isotherm models (equations, parameters and data points), microscopy images, size distributions by static light scattering, protein adsorption data, additional IR data and further experimental details. See DOI: https://doi.org/10.1039/d6na00243a.
