# Supplementary material for: Surface complexation and multilayer formation in the adsorption of NADA and phosphate on magnetic iron oxide nanoparticles: implications for bioseparation
Source: Nanoscale Adv. 2026 Jun 25. Online ahead of print. doi: 10.1039/d6na00243a (PMC13325004; doi:10.1039/d6na00243a)
Supplement: NA-OLF-D6NA00243A-s001 [file NA-OLF-D6NA00243A-s001.pdf]

Supplementary Information (SI) for Nanoscale Advances.
This journal is © The Royal Society of Chemistry 2026

Supplementary Material

Surface complexation and multilayer formation in the adsorption of NADA and phosphate on magnetic iron oxide nanoparticles: Implications for bioseparation

Paula Fraga-García[a], Carlos Eduardo Díaz-Cano[a], Spartak S. Khutsishvili[b,c], Vanessa Jurado-Davila[d], Lucía Abarca-Cabrera[a], Jozef Lengyel[d]*

[a] Technical University of Munich (TUM), School of Engineering and Design, Department of Energy and Process Engineering, Chair of Bioseparation Engineering, Boltzmannstraße 15, 85748, Garching, Germany.
[b] School of Medicine, Georgian American University, 10 M. Aleksidze St., 0160 Tbilisi, Georgia
[c] Rafael Agladze Institute of Inorganic Chemistry and Electrochemistry, Ivane Javakhishvili Tbilisi State University, 11 E. Mindeli St., 0186 Tbilisi, Georgia
[d] Technical University of Munich (TUM), School of Natural Sciences, Department of Chemistry, Chair of Physical Chemistry, Lichtenbergstraße 4, 85748, Garching, Germany

Table S1. Composition of modified ASW media

Chemical	Concentration
NaCl	27 g L^{-1}
$MgSO_4 \cdot 7\ H_2O$	6.6 g L^{-1}
$CaCl_2 \cdot 2\ H_2O$	1.5 g L^{-1}
KNO_3	1 g L^{-1}
KH_2PO_4	0.07 g L^{-1}
$FeCl_3 \cdot 6\ H_2O$	0.014 g L^{-1}
$Na_2EDTA \cdot 2\ H_2O$	0.021 g L^{-1}
Micronutrients solution	1 mL L^{-1}
Chemicals of the micronutrients solution	
$ZnCl_2$	0.04 g L^{-1}
H_3BO_3	0.6 g L^{-1}
$CoCl_2 \cdot 2\ H_2O$	0.04 g L^{-1}
$MnCl_2 \cdot 4\ H_2O$	0.629 g L^{-1}
$(NH_4)_6Mo_7O_{24} \cdot 4\ H_2O$	0.37 g L^{-1}

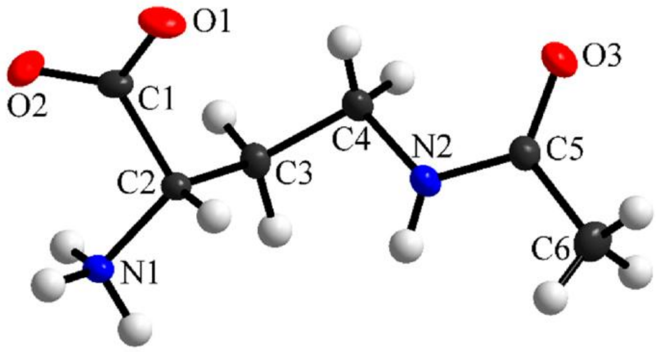

Figure S1. Schematic illustration of the chemical structure of N-γ-acetyl-L-2,4-diaminobutyric acid (γ-NADA). Reproduced from Martin *et al*. 2020, ref. 14, DOI:10.3390/cryst10121136, under the terms of the CC BY 4.0 license (https://creativecommons.org/licenses/by/4.0/).

Equations of adsorption isotherms

$$q_e = \frac{Q_{max} R_L C_e}{1+k_L C_e} \qquad \text{(Langmuir)}$$

$$q_e = k_F C_e^{1/n} \qquad \text{(Freundlich)}$$

$$q_e = \frac{K_r C_e}{1+a_R C_e^{\beta}} \qquad \text{(Redlich-Peterson)}$$

where q_e (mg·g^{-1}) is the equilibrium adsorption amount at equilibrium concentration of C_e (mg·L^{-1}); Q_{max} is the maximum capacity of the adsorbent (mg·g^{-1}); R_L is the separation factor, and k_L is the Langmuir adsorption constant (L·mg^{-1}). k_F (mg^{1-n}·L^{n}·g^{-1}) and n are the Freundlich affinity coefficient and linearity, respectively. K_r is the Redlich-Peterson adsorption constant (L·mg^{-1}), a_R is a Redlich–Peterson isotherm constant (L·mg^{-1}), β is Redlich–Peterson isotherm exponent.

Table S2. Isotherm data for impure NADA

Equilibrium concentration, g L^{-1}	Load, g g^{-1}	Freundlich, g g^{-1}	Langmuir, g g^{-1}	Redlich-Peterson, g g^{-1}
30.6855	0.16165	0.15955	0.14071	0.14558
15.66836	0.089	0.11419	0.12281	0.12103
9.16925	0.09115	0.08746	0.1037	0.10019
4.17215	0.12807	0.0591	0.07156	0.06952
2.2581	0.02963	0.04354	0.04828	0.0482
1.14532	0.01156	0.03106	0.02859	0.02969

0.55525	0.00926	0.02166	0.01521	0.01643
0.28095	0.00397	0.01543	0.00806	0.00895
0.15174	0	0.01136	0.00445	0.00503
0.08536	0	0.00853	0.00254	0.0029

Table S3. Isotherm parameters for impure NADA

**Freundlich**	**Langmuir**	**Redlich-Peterson**
k_f = 0.0290	Q_{max} = 0.1659 g g^{-1}	k_r= 0.0352 L g^{-1}
n= 2.0091	R_L= 0.9356<br>k_L= 0.1817 L g^{-1}<br>k_D=5.50 g L^{-1}	β= 0.8786<br>a_R= 0.3167
R^2= 0.7594	R^2= 0.8610	R^2= 0.7926

Table S4. Isotherm data for pure NADA

**Equilibrium concentration, g L^{-1}**	**Load, g g^{-1}**	**Freundlich, g g^{-1}**	**Langmuir, g g^{-1}**	**Redlich-Peterson, g g^{-1}**
0.2432	0.00306	0.02567	0.01042	0.0068
0.49387	0.00625	0.03545	0.02034	0.01379
0.97996	0.01686	0.04844	0.03749	0.02727
1.99722	0.01726	0.06701	0.06653	0.05484
3.93919	0.12315	0.09131	0.10526	0.10314
7.7515	0.18284	0.12431	0.14918	0.17343
15.56395	0.21452	0.17079	0.19039	0.22265
31.82799	0.1894	0.23662	0.22139	0.1862

Table S5. Isotherm parameters for pure NADA

Freundlich	Langmuir	Redlich-Peterson
K_f = 0.0489	Q_{max} = 0.2623 g g^{-1}	k_r= 0.028 L g^{-1}
n= 2.1944	R_L= 0.9211<br>k_L=0.1702 L g^{-1}<br>k_D=5.87 g L^{-1}	β= 1.9231<br>a_R=0.0049
R^2= 0.7771	R^2= 0.9410	R^2= 0.9642

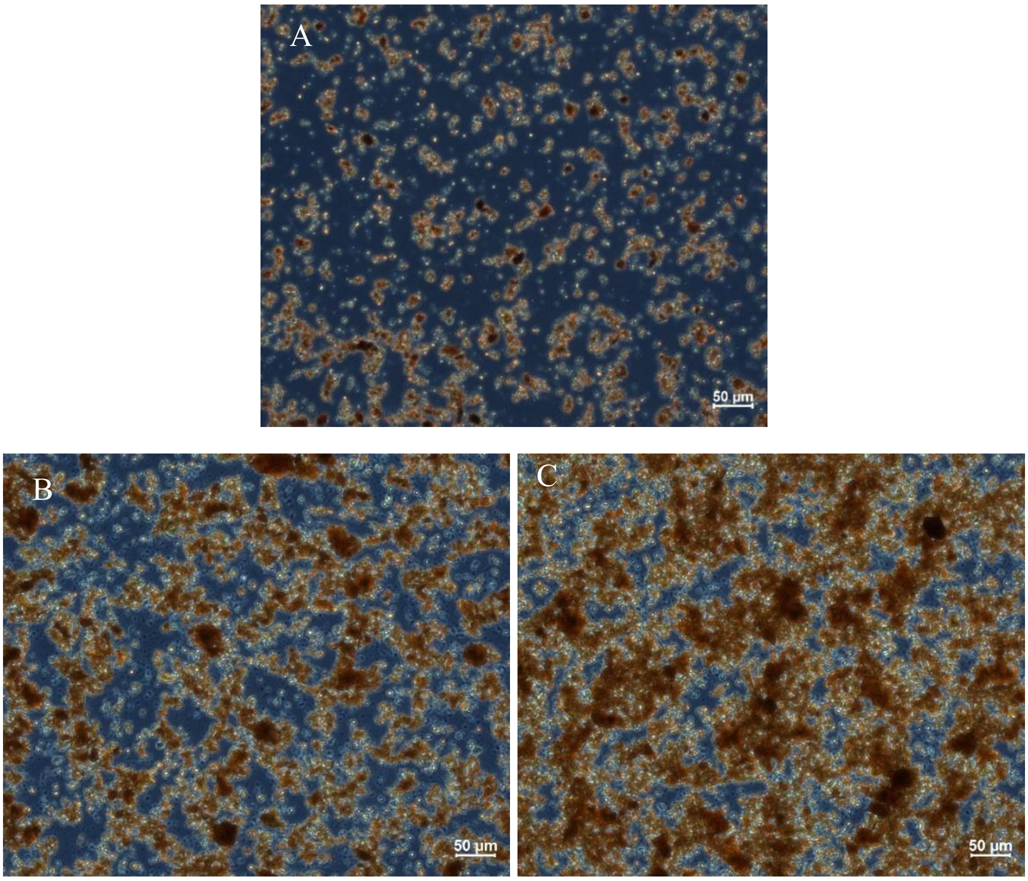

Figure S2. Phase contrast images of BIONs incubated with different NADA concentrations (20x; 10 g_{BION} L^{-1}). A: BIONs; B: BIONs incubated with 22.5 g_{NADA} L^{-1}; C: BIONs incubated with 70 g_{NADA} L^{-1}.

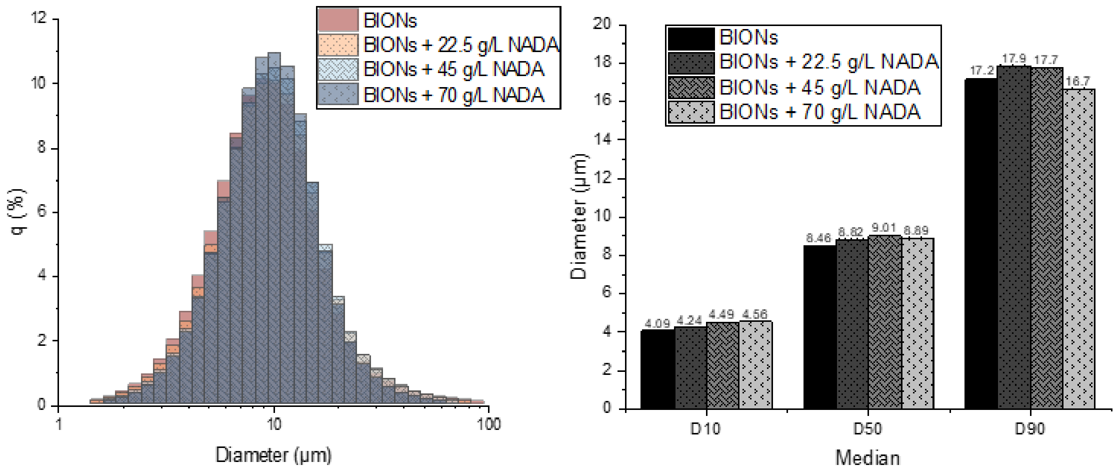

Figure S3. Size distributions (left) and median size values (right) of BIONs and BIONs incubated with different NADA concentrations

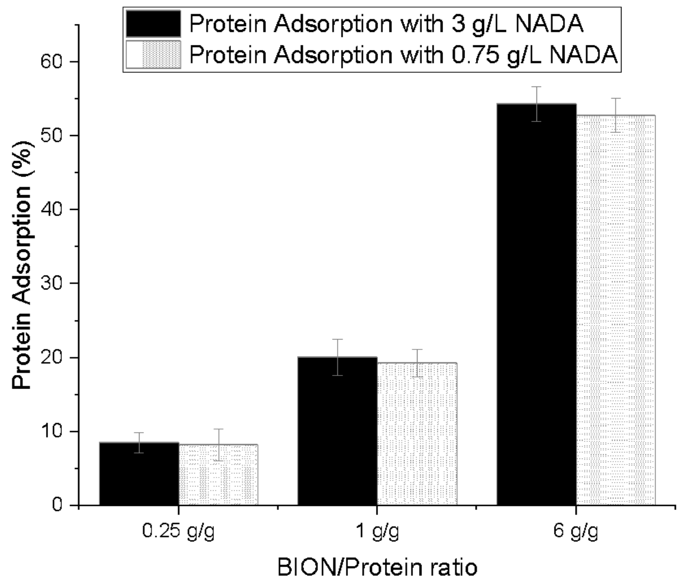

Figure S4. Protein adsorption onto BIONs incubated with *M. salina* lysate for two initial NADA concentrations in solution and for different BION-to-protein mass ratios.

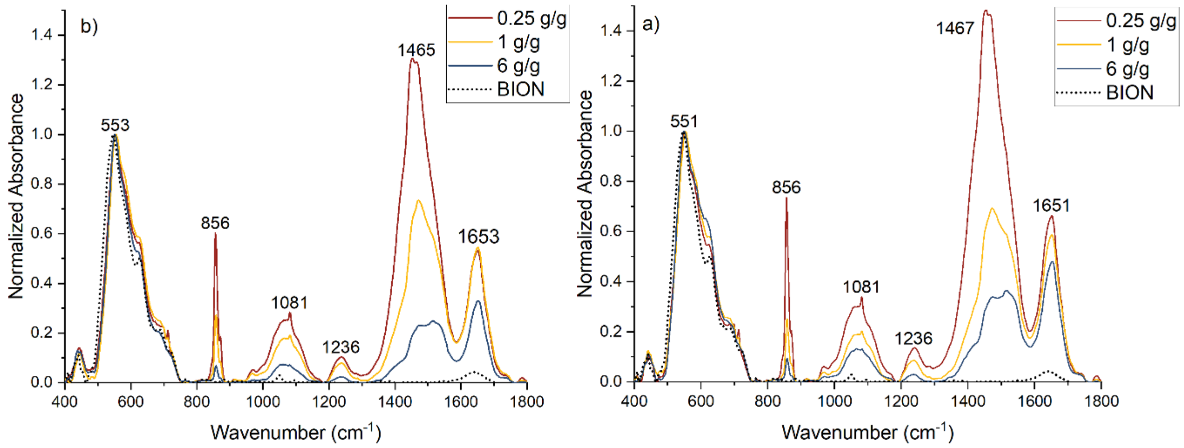

Figure S5. FTIR spectra of BIONs incubated with *M. salina* lysates complemented with 0.75 g L^{-1} NADA (left) and 3.0 g L^{-1} NADA (right).

Table S6. Isotherm data for phosphate in water

Equilibrium concentration, mg L^{-1}	Load, mg g^{-1}	Freundlich, mg g^{-1}	Langmuir, mg g^{-1}	Redlich-Peterson, mg g^{-1}
691.66188	9.38792	11.46109	10.76966	9.29886
459.2146	10.02426	9.89497	10.23972	10.77887
205.3323	11.43834	7.41336	8.67011	10.82916
88.44678	7.40821	5.48022	6.34518	6.9222
54.74454	2.2861	4.6138	4.91874	4.68347
23.13003	2.02292	3.38713	2.72297	2.09857
6.33861	1.81866	2.12891	0.89343	0.58463
2.56773	1.05428	1.53948	0.3787	0.23722
1.02914	0.7563	1.10899	0.15471	0.09511
0.66737	0.30623	0.94939	0.10078	0.06168
0.0656	0.20025	0.41307	0.00998	0.00606
0	0.08304	0	0	0
0.02121	0.0414	0.27551	0.00323	0.00196

Table S7. Isotherm parameters for phosphate in water

Freundlich	Langmuir	Redlich-Peterson
k_f = 1.098	Q_{max} = 11.99 mg g^{-1}	k_r= 0.094 L mg^{-1}
n= 2.7876	R_L= 0.8956<br>k_L=0.0127L mg^{-1}	β= 1.6921<br>a_R=0.0001
	k_D=78.74 mg L^{-1}	
R^2= 0.8524	R^2= 0.9094	R^2= 0.9561

Table S8. Isotherm data for phosphate in ASW

Equilibrium concentration, mg L^{-1}	Load, mg g^{-1}	Freundlich, mg g^{-1}	Langmuir, mg g^{-1}	Redlich-Peterson, mg g^{-1}
626.81407	5.55744	10.86144	10.34104	9.12014
403.4073	9.88206	10.47698	10.3351	9.49476
165.9770	14.03922	9.74307	10.31134	10.27109
57.91487	12.17881	8.9393	10.23674	11.15312
12.64913	9.58695	7.89355	9.84521	11.67287
1.03747	5.96738	6.43366	6.36118	5.57334
0.08324	1.92127	5.23434	1.17383	0.65111
0	1.30209	0	0	0
0	0.7512	0	0	0
0	0.45027	0	0	0
0	0.22167	0	0	0
0	0.12595	0	0	0

Table S9. Isotherm parameters for phosphate in ASW

Freundlich	Langmuir	Redlich-Peterson
k_f = 5.414	Q_{max} =10.352 mg g^{-1}	k_r= 8.071 L mg^{-1}
n= 12.229	R_L= 0.9401<br>k_L=1.5365 L mg^{-1}<br>k_D=0.65 mg L^{-1}	β= 1.094<br>a_R=0.4826
R^2= 0.7425	R^2= 0.8467	R^2= 0.8331

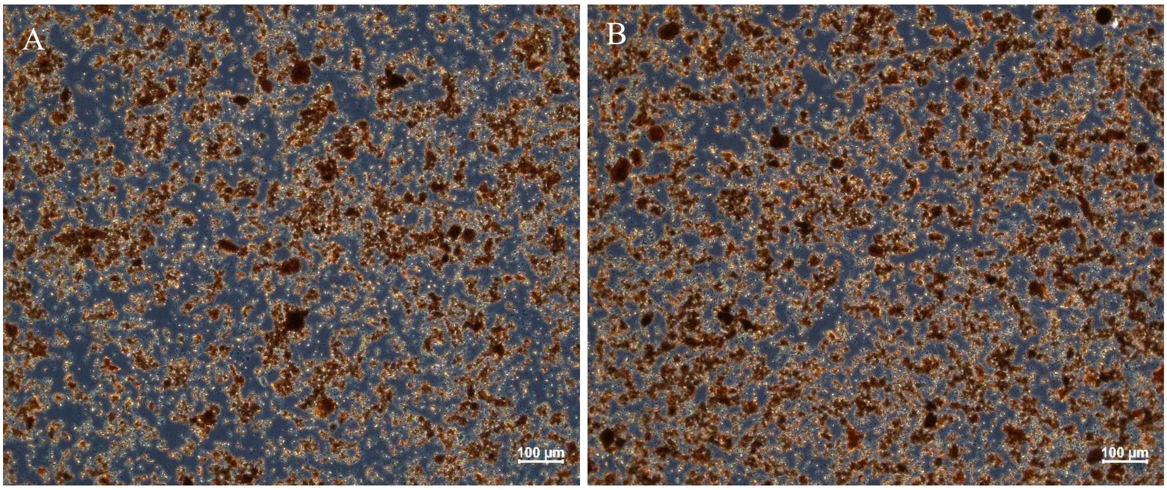

Figure S6. Phase contrast images of BIONs (A) and BIONs incubated with 32 mg_{PO4} L^{-1} in ddH20 (10x; 10 g_{BION} L^{-1}).

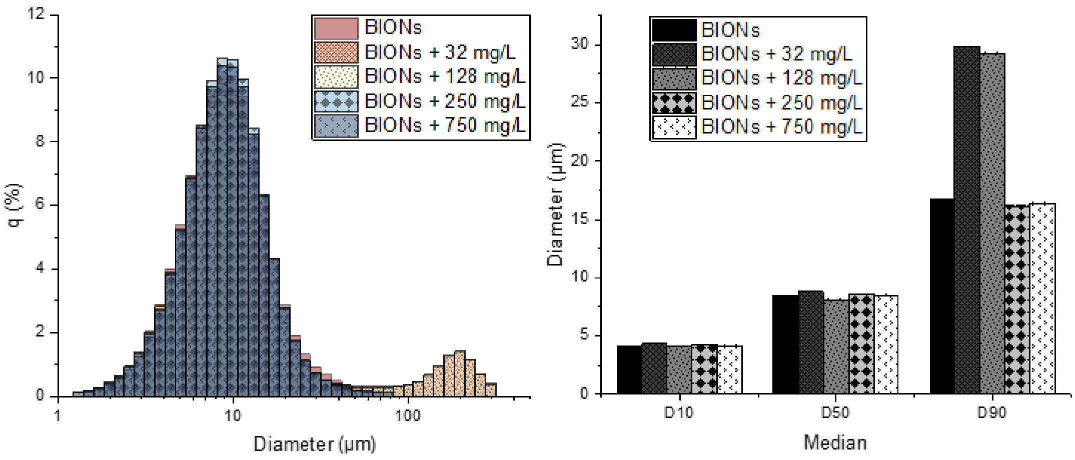

Figure S7. Size distribution (left) and median size values (right) of BIONs and BIONs incubated with different PO4 concentrations in ddH20.

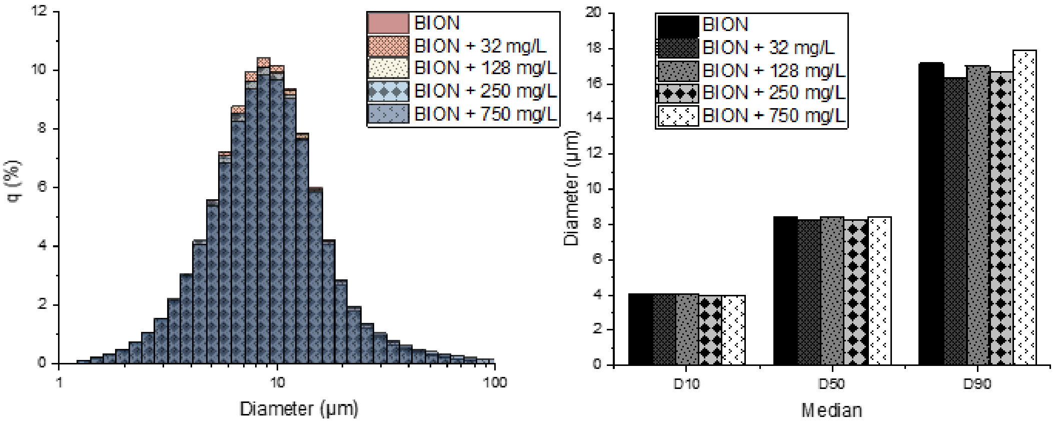

Figure S8. Size distribution (left) and median size values (right) of BIONs and BIONs incubated with different PO4 concentrations in ASW.

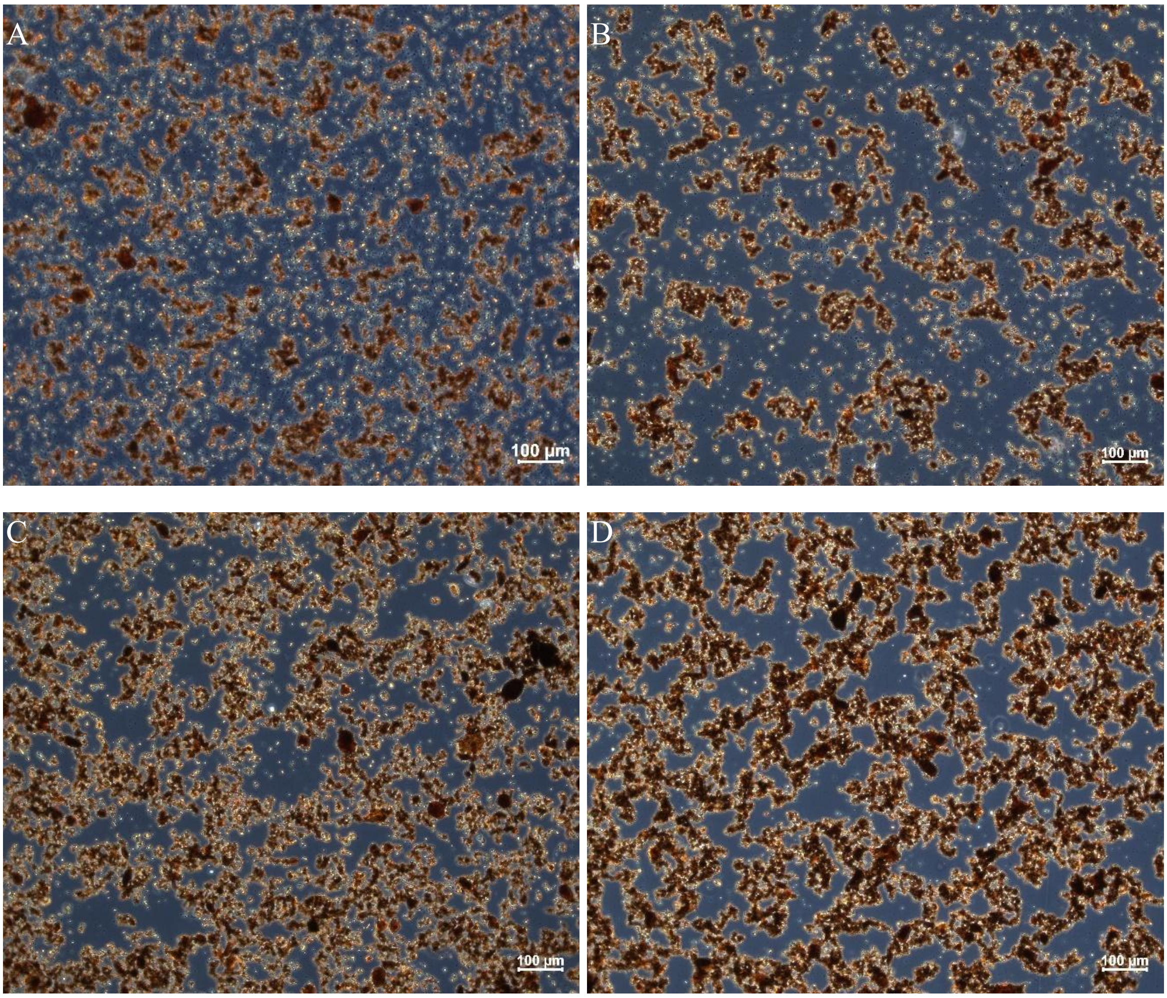

Figure S9. Phase contrast images of BIONs (A), BIONs incubated with 32 mg_{PO4} L^{-1} (B), with 128 mg_{PO4} L^{-1} (C) and with 750 mg_{PO4} L^{-1} (D) in ASW (10x; 10 g_{BION} L^{-1}).

Reference

L. Martin, W. Klein, S. P. Schwaminger, T. F. Fässler and S. Berensmeier, Crystal Structure and Spectroscopic Analysis of the Compatible Solute Nγ-Acetyl-L-2,4-Diaminobutyric Acid, *Crystals*, 2020, **10**, 1136
